# Mucus-derived glycans are inhibitory signals for *Salmonella* Typhimurium SPI-1-mediated invasion

**DOI:** 10.1016/j.celrep.2025.116304

**Published:** 2025-09-23

**Authors:** Kelsey M. Wheeler, Michaela A. Gold, Corey A. Stevens, Karsten Tedin, Amanda M. Wood, Deniz Uzun, Gerardo Cárcamo-Oyarce, Bradley S. Turner, Marcus Fulde, Jeongmin Song, Jessica R. Kramer, Katharina Ribbeck

**Affiliations:** 1 Biological Engineering Department, Massachusetts Institute of Technology, Cambridge, MA, USA; 2 Microbiology Program, Massachusetts Institute of Technology, Cambridge, MA, USA; 3 Institute of Microbiology and Epizoonotics, Freie Universität Berlin, Berlin, Germany; 4 Department of Bioengineering, University of Utah, Salt Lake City, UT, USA; 5 Department of Microbiology and Immunology, Cornell University, Ithaca, NY, USA; 6 These authors contributed equally; 7 Lead contact

## Abstract

Mucus forms a critical barrier against enteric pathogens like *Salmonella enterica* serovar Typhimurium. While *in vivo* studies indicate that secreted, gel-forming mucins and specifically core 3 glycosylation are protective against *S*. Typhimurium, the molecular mechanisms involved remain unclear. Here, we demonstrate that native intestinal mucins inhibit *Salmonella* invasion of colonic epithelial cells by downregulating the type 3 secretion system through suppression of the key virulence regulator, HilD. Our study identifies mucin glycans and specific mucin sugars, namely N-acetyl galactosamine and N-acetyl glucosamine, as the components responsible for mucin’s anti-virulence effect, likely via functional or direct interaction with HilD’s putative carbohydrate-binding domain. Notably, we find that the native presentation of these sugars is important for activity. These insights provide a mechanistic foundation for mucin-based strategies to combat enteric infections and, given the prevalence of homologous AraC-type regulators in other pathogens, suggest mucins’ potential as broad-spectrum anti-virulence agents.

## In brief

Host mucins and synthetic glycopolypeptides block *Salmonella* virulence via glycan-mediated suppression of *Salmonella* pathogenicity island 1 (SPI-1). In this study, Wheeler et al. reveal that gastrointestinal mucins and their glycans suppress *Salmonella* invasion by downregulating SPI-1 via HilD. Synthetic glycopolypeptides displaying mucin sugars recapitulate this anti-virulence effect, offering new insights into glycan-based anti-infective strategies.

## Graphical Abstarct

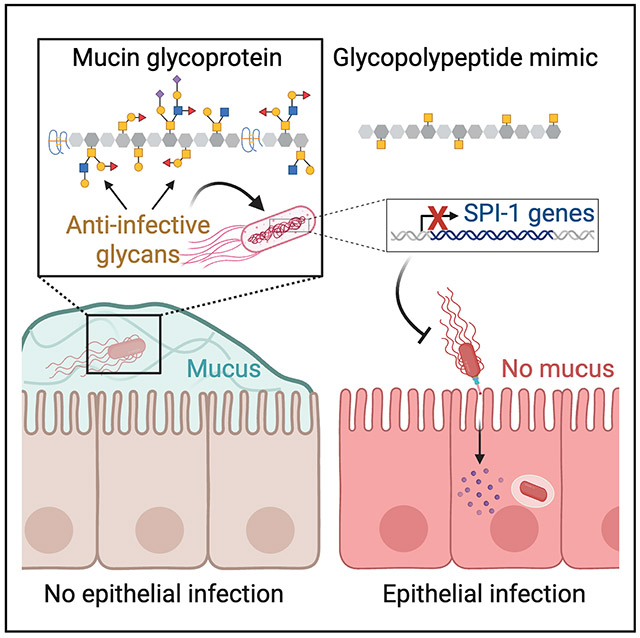

## INTRODUCTION

Mucus is the first line of defense against enteric infections caused by bacterial pathogens. A key protective component of intestinal mucus is the secreted mucin glycoprotein, MUC2, which has been shown to limit pathogen burden and infection severity for *Citrobacter rodentium* and *Salmonella enterica* serovar Typhimurium.^1,2^ Mucus architecture varies significantly along the gastrointestinal tract. The small intestine has a loose, continuous mucus layer,^3,4^ while the mouse cecum lacks a complete layer, exposing the epithelium.^5^ In the colon, mucus is divided into two layers: an outer layer harboring bacteria and a mostly sterile inner layer of dense mucus that serves as a barrier to the underlying tissue.^6,7^

*S.* Typhimurium is a highly gut-adapted pathogen exhibiting sophisticated molecular mechanisms for colonizing the intestinal epithelium. In particular, *Salmonella* pathogenicity island 1 (SPI-1) is a gene cluster encoding a type 3 secretion system (T3SS), a key virulence factor that facilitates irreversible docking to host cells and invasion.^8-11^ While this bacterium can penetrate the small intestine’s loose mucus and accumulate near the villi, *S*. Typhimurium initiates infection in regions with reduced mucus density, such as the mouse cecum or Peyer’s patches.^4,5,12,13^ The preference of *S*. Typhimurium to invade tissues with less mucus, even when the mucus is loose and penetrable, hints at the presence of additional anti-infective mechanisms within the mucus beyond its barrier functions. Notably, the T3SS was found to play a more important role in mice lacking MUC2 than in wild-type (WT) mice, suggesting that MUC2 may impact the SPI-1 virulence system.^2^ Moreover, core 3 *O*-glycans, consisting of N-acetyl glucosamine β1-3 linked to N-acetyl galactosamine on serine or threonine (GlcNAc-β1-3GalNAcα-Ser/Thr), were important for controlling *Salmonella*-induced disruption of epithelial barrier integrity, whereas Muc2 protein and its remaining glycosylation are crucial for controlling *S*. Typhimurium burden and overt intestinal pathology.^2^ Together, these studies underscore that mucus protects the host through both physical and chemical strategies, whereby at high densities, mucus can physically impede bacterial diffusion and motility, while at lower densities or in looser regions, chemical interactions, such as glycan-mediated suppression of virulence gene expression, may provide an additional layer of defense. We posit that these mechanisms act in concert to prevent enteric infections. However, the specific contributions of mucin glycans to chemical inhibition of *Salmonella* virulence remain incompletely defined. To fill this gap, we have systematically dissected the anti-infective actions of MUC2 on *S*. Typhimurium.

## RESULTS

### Gastrointestinal mucins attenuate *S*. Typhimurium host cell invasion

Invasion of host epithelial cells is a hallmark of *S*. Typhimurium pathogenesis and a prerequisite for triggering inflammation and systemic dissemination.^14,15^ To determine the protective role of mucin in this process, we purified the primary intestinal mucin, MUC2, from porcine intestines and reconstituted it in SPI-1 inducing medium. Scanning electron microscopy revealed a dense mucin matrix in which *S*. Typhimurium appeared interspersed throughout (Figures 1A and S1A), illustrating the structural context of the mucin environment and highlighting the availability of potential interaction sites.

To assess the functional impact of the mucin environment, we quantified *S*. Typhimurium invasion of HT-29 monolayers, an undifferentiated human colorectal adenocarcinoma epithelial cell line that does not produce mucin.^16,17^ When HT-29 cells were cultured in medium with exogenous MUC2 and infected with either the MUC2-treated *S*. Typhimurium LT2 (attenuated virulence *in vivo*) strain^18^ or 14028s (virulent) strain,^19^ fewer bacteria invaded the epithelial cells (Figures 1B and S1B). This inhibitory effect was maintained in polarized HT-29 cells grown on transwell inserts, indicating that mucin-mediated protection is independent of epithelial polarization (Figures S1C and S1D).

Notably, invasion inhibition was not restricted to intestinal mucin. MUC5AC, a gastric mucin purified from porcine stomach, similarly suppressed epithelial invasion by both *S*. Typhimurium strains (Figures 1C and S1B). Confocal microscopy confirmed these findings, showing reduced invasion and perinuclear localization of bacteria in MUC2-treated cultures (Figures 1D and S1E). Together, these results demonstrate that mucins from gastrointestinal sources closely associate with *S*. Typhimurium and, in turn, suppress its ability to invade epithelial cells despite differences in their glycosylation patterns.^20,21^

### Gastrointestinal mucins downregulate the SPI-1

*S.* Typhimurium pathogenicity largely arises from SPIs, which encode virulence factors critical for host cell invasion and intracellular survival.^10,11^ After establishing contact with epithelial cells, *S*. Typhimurium utilizes its T3SS to inject bacterial effector proteins directly into host cells, facilitating cytoskeletal remodeling and bacterial uptake.^8-11^

To better understand the molecular basis of mucin-mediated invasion inhibition, we tested whether mucins modulate expression of SPI-1 genes, which encode the T3SS. Quantitative polymerase chain reaction (qPCR) revealed that MUC2 triggered a dose-dependent decrease in the expression of two SPI-1 genes, SPI-1 regulator *hilA* and T3SS-1 needle complex gene *prgH,* for both LT2 and 14028s strains (Figure 2A). To determine the extent of MUC2-mediated gene expression changes, we performed RNA sequencing on LT2 cultured with and without MUC2 in the absence of host cells. Purified MUC2 significantly downregulated 190 genes (false discovery rate < 0.05) and the entirety of SPI-1, whereas 96 genes were upregulated >2-fold (Figure 2B). MUC5AC similarly downregulated the SPI-1 regulators *hilA* and *invF,* the needle complex gene *prgH,* and the effector gene *sopE2* (Figure 2C)*.* Pathway analysis revealed an enrichment of *Salmonella* infection genes among the downregulated gene set as well as an enrichment of multiple metabolic pathways, both the upregulated and downregulated sets (Figure S2A). This suggests that MUC2 triggers a global metabolic shift in addition to reducing SPI-1 expression. Although adding mucin to SPI-1-inducing medium did not alter growth (Figure 2D), *S.* Typhimurium could utilize MUC2 as its sole carbon source in M9 medium, demonstrating its capacity to metabolize mucin components (Figure S2B).

To determine whether MUC2-mediated transcriptional suppression of SPI-1 genes translated to reduced SPI-1 protein expression, we performed data-independent acquisition (DIA)-based proteomics on *S.* Typhimurium LT2 cultured under SPI-1-inducing conditions, with or without exogenous MUC2. Across the proteome, we identified 1,730 bacterial proteins, including nine SPI-1 components (Figure 2E). Consistent with our RNA-sequencing data, all nine SPI-1 proteins were downregulated in the presence of MUC2 (Figures 2E and S2C). These proteins included both structural elements of the T3SS needle complex and secreted effector proteins (Figure S2C). Because proper T3SS function requires a balanced production of its structural and effector components, the coordinated downregulation of these proteins suggests that MUC2 treatment disrupts the secretion system assembly and activity. This, in turn, likely impairs effector translocation into host cells, thereby blocking the cytoskeletal remodeling required for invasion. Taken together, these findings support a model in which MUC2 suppresses SPI-1 gene expression, leading to reduced T3SS protein synthesis and ultimately inhibiting *S*. Typhimurium invasion of epithelial cells.

Given the conserved function of MUC2 and MUC5AC in suppressing *Salmonella* invasion and SPI-1 gene expression, we next sought to test whether a large anionic polymer alone would be sufficient to achieve similar effects. To this end, we evaluated carboxymethyl cellulose (CMC), a high molecular weight, anionic polysaccharide commonly used as a mucin mimic for its viscosity and physical barrier properties but lacking glycan-specific biochemical features. Notably, CMC did not inhibit invasion (Figure S3A) or suppress SPI-1 gene expression (Figure S3B), suggesting that mucin-mediated suppression of epithelial invasion is driven by specific biochemical interactions rather than nonspecific steric hindrance alone.

### Mucin glycans are anti-infective cues

**Figure 3.**
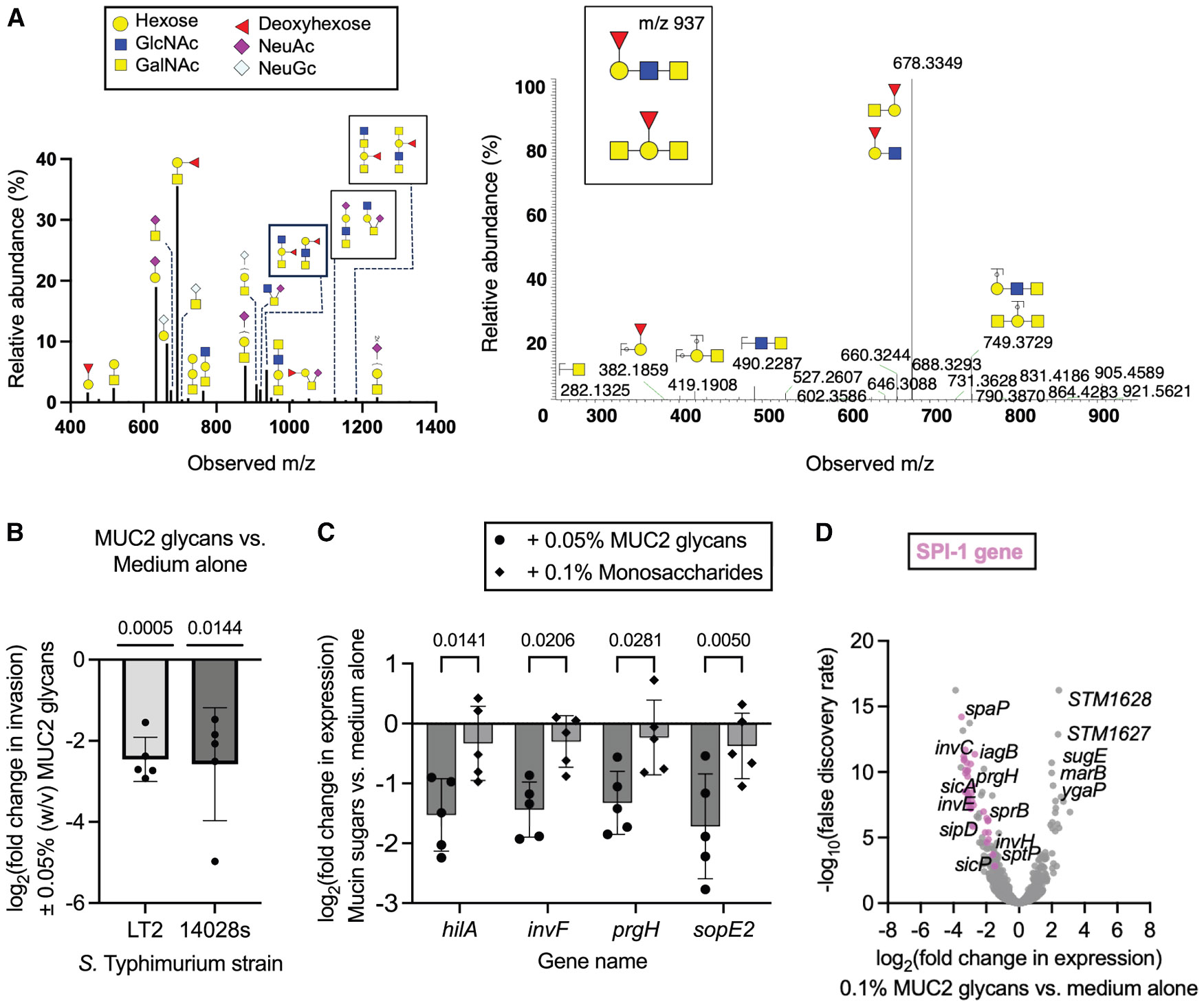
MUC2-derived glycans drive *S.* Typhimurium virulence attenuation (A) MUC2 glycosylation patterns. Left: MS spectra of permethylated porcine MUC2 O-glycans. Data are provided as an average of liquid chromatography-mass spectrometry spectra and are deconvoluted. Masses are shown as M + Na. Structures assigned to peaks were hand-annotated with predicted compositions based on known biosynthetic pathways and fragmentation patterns. See Table S1 for full assignments. Right: example MS/MS fragmentation, m/z 937, via collision-induced dissociation, and is an average of multiple spectra. Fragmentation indicates both core 1 and core 3 structures are present. (B) Host-cell invasion, as determined by a gentamicin protection assay of *S*. Typhimurium grown with a MUC2 glycan pool (0.05% w/v), of HT-29 cells infected at an MOI of 20. A one-sample t test was performed to evaluate whether the change in invasion was significantly different than 0. Exact *p* values are shown above each bar. (C) Effect of mucin glycans (0.05% w/v, ∼0.9 mM, assuming a molar mass of 565 Da, i.e., molar mass of the fucosylated core 1 structure) or pool of monosaccharides (0.1% w/v total combined weight, corresponding to 0.02% w/v or ∼0.9–1.2 mM of each monosaccharide) on SPI-1 gene expression for *S.* Typhimurium 14028s, as measured by qPCR. A two-way ANOVA was conducted to evaluate the effect of MUC2 concentration and *Salmonella* strain on SPI-1 gene expression. There was a significant effect of MUC2 concentration on SPI-1 expression. Exact *p* values reported. (D) Gene expression changes, as determined by RNA sequencing, of *S.* Typhimurium LT2 cultured with MUC2 glycans (0.1% w/v) relative to medium alone (*n* = 3 biologically independent replicates). Individual points represent different genes in the *Salmonella* genome. SPI-1 genes are denoted in pink. In (B) and (C), data points represent individual biological replicates, bars represent mean log_2_-adjusted changes relative to medium alone, and error bars represent the SD. See also Figures S1B and ^S4^, Table S1.

Given the relevance of the core 3 glycan in controlling *S.* Typhimurium damage of mouse intestinal epithelium,^2^ we hypothesized that mucin-derived glycans might be a signaling component responsible for SPI-1 inhibition by mucin. To test this possibility, we isolated complex porcine MUC2 *O*-glycans by non-reductive alkaline β-elimination ammonolysis. Profiling of the resulting glycan pool by mass spectrometry (MS) identified 38 complex carbohydrate peaks at >0.1% relative abundance (Figure 3A; Table S1). Most *O*-glycans were consistent with core 1 (Gal-β1-3GalNAcα-) and core 3 (GlcNAc-β1-3GalNAcα-) structures, which were observed as compositional/positional isomers by chromatography and MS/MS fragmentation. Multiple sialic-acid-containing (N-acetylneuraminic acid and N-glycolylneuraminic acid) and fucose-containing glycoforms and fragments were present, as well as a complex mix of trace highly complex glycans with m/z > 1,600, potentially corresponding to dozens of glycoform compositions and positional isomers. We then exposed *S.* Typhimurium to this complex glycan pool and measured host-cell invasion for LT2 or 14028s. MUC2 glycans were sufficient to inhibit HT-29 cell invasion (Figures 3B and S1B), indicating that these complex sugars may be responsible for mucin’s antiinvasion activity. The MUC2 glycan pool, but not a pool of the component monosaccharides, suppressed SPI-1 gene expression (Figure 3C). Thus, we suspect that the precise arrangement and presentation of these sugars are important to their function.

To further clarify the virulence-neutralizing function of mucin-derived glycans, we evaluated the transcriptional profile of *S.* Typhimurium LT2 following exposure to media with or without the MUC2 glycans pool by RNA-sequencing. MUC2 glycans upregulated 69 genes more than 2-fold and downregulated 154 genes, including all SPI-1 genes (Figure 3D). We compared the overlapping differential expression profiles for *S*. Typhimurium following exposure to MUC2 or MUC2 glycans relative to medium alone to determine which portions of the mucin response were specific to the intact glycoprotein or were glycan-dependent. Pathway analysis identified *Salmonella* infection genes as significantly enriched within the overlapping downregulated gene sets (Figure S4A), providing further evidence that SPI-1 suppression is glycan-dependent. Among the upregulated genes, only eight genes were identified in both the MUC2 and MUC2 glycan profiles, and distinct metabolic pathways were enriched (Figure S4B). Together, these results suggest that while the suppressive effects on *Salmonella* infection pathways are conserved, mucin and liberated glycans drive distinct metabolic processes.

### Mucin sugars inhibit SPI-1 through HilD

**Figure 4.**
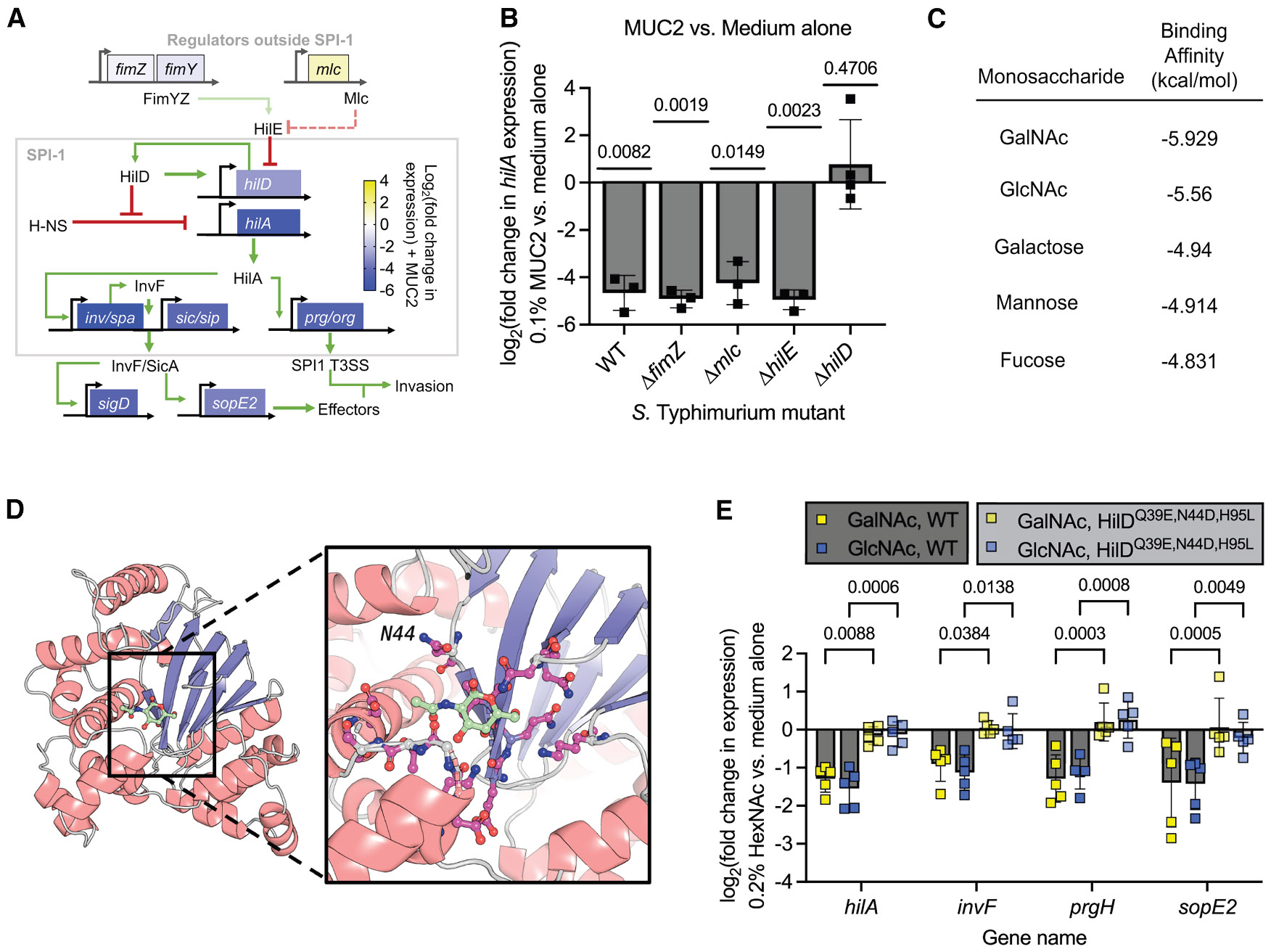
MUC2-mediated inhibition of *S*. Typhimurium virulence is HilD-dependent (A) Schematic illustrating how MUC2 inhibits gene expression of SPI-1-encoded regulators and downstream genes but not regulators encoded outside of SPI-1. Gene expression changes were determined by RNA sequencing (Figure 1E) with MUC2 (0.1% w/v) relative to medium alone, overlaying the SPI-1 regulatory network.^15^ (B) Effect of MUC2 (0.1% w/v) on *hilA* gene expression in different mutant backgrounds or the *S.* Typhimurium parent strain 14028s (WT), as measured by qPCR. A one-sample t test was performed to evaluate whether the change in expression was significantly different than 0. Exact *p* values are shown above each bar. (C) Autodock Vina predicted binding affinities for monosaccharides to HilD. Docking scores were calculated for individual monosaccharides using the predicted structure of Salmonella HilD (modeled with trRosseta). Ligands were docked into the putative carbohydrate-binding pocket (identified with PocketMiner) using AutoDock Vina, and binding affinities (kcal/mol) are reported. (D) Predicted binding pose of GalNAc within the HilD ligand-binding pocket. Binding location and pose were visualized using PyMOL. Highlighted residues indicate candidate interaction points, including N44 (previously implicated in gut-signal sensing with Q39 and H95), suggesting this region may play a role in glycan sensing. (E) Effect of HilD binding site mutations on SPI-1 gene expression, measured by qPCR, in response to GalNAc or GlcNAc (0.2% w/v, 9.0 mM). A two-way ANOVA with Tukey’s multiple comparison test was conducted to evaluate the effect of HilD mutations on HexNAc-mediated suppression of SPI-1 gene expression. Exact *p* values are reported. In (B) and (E), data points represent individual biological replicates, bars represent mean log_2_-adjusted changes relative to medium alone, and error bars represent the SD. See also Figures S5 and ^S6^.

*Salmonella* tightly regulates SPI-1 gene expression through a complex network of factors to optimize virulence.^15,22-27^ Notably, transcriptomic analysis revealed that MUC2 differentially regulates several of these factors in addition to the central SPI-1 regulator HilD and its downstream SPI-1 encoded genes (Figure 4A). Given the differential regulation of metabolic pathways in the presence of mucins, we hypothesized that a global regulator like Mlc, which was also slightly upregulated by MUC2 and controls sugar uptake, metabolism, and SPI-1 expression,^23^ might be involved in sensing these host glycans. Alternatively, the FimYZ two-component system, which was slightly downregulated by MUC2 and known to control SPI-1,^24^ could play a role. For both of these regulatory systems, their effects on SPI-1 are modulated by an interaction between HilE and HilD.^23-25^

To determine if any of these components were involved in MUC2 sensing, we measured *hilA* gene expression, a key downstream activator of the SPI-1 system and a sensitive readout of SPI-1 expression, in a series of 14028s deletion mutants. These regulatory mutants were chosen based on their known roles in SPI-1 control, with Δ*fimZ* and Δ*mlc* strains lacking regulators that influence SPI-1 indirectly through HilD; the Δ*hilE* strain lacking a repressor that directly binds and inhibits HilD, leading to elevated SPI-1 expression under certain conditions, and the Δ*hilD* strain lacking the master regulator that integrates multiple environmental signals to activate SPI-1 genes. While MUC2 retained its ability to suppress *hilA* in *S.* Typhimurium Δ*fimZ*, Δ*mlc*, and Δ*hilE* mutants, it was impaired in the Δ*hilD* background (Figure 4B), indicating its potential relevance to the mucin response.

HilD is a putative AraC/XylS-like transcriptional regulator that can sense various small-molecule signals.^28-32^ Consistent with prior studies,^29^ structural modeling of HilD using trRosetta^33^ and binding pocket identification with PocketMiner^34^ predicted a putative N terminal “jelly-roll” or cupin-like motif with homology to carbohydrate-binding proteins and found in AraC-like DNA-binding proteins in other pathogens, including *Shigella flexneri,* enterotoxigenic *Escherichia coli,* and *Vibrio cholerae*.^35^ Notably, the predicted ligand binding pocket was a relatively shallow cleft between a β-sheet and an α-helix, characteristic of the jelly roll fold, and contained several charged and polar residues (Figures S5A and S5B).

Given the homology of the putative binding pocket to known carbohydrate-binding domains, we hypothesized that the HilD jelly-roll fold may directly interact with mucin-derived glycans. Since HilD is localized in the cytosol and unlikely to encounter fully intact mucin polymers, we focused our analysis on simple sugar structures. To explore potential interactions between mucin sugars and the predicted HilD ligand binding domain, we performed a virtual screen of a monosaccharide library using AutoDock Vina.^36,37^ Ligand docking simulations identified N-acetyl-D-galactosamine (GalNAc) and N-acetyl-D-glucosamine (GlcNAc) as potential ligands for HilD (Figure 4C). As expected for carbohydrate-protein interactions, the predicted affinities were low, consistent with the promiscuous and low-affinity (K_d_ ≈ 10^−3^ M) nature of carbohydrate recognition domains.^38-40^

These *in silico* results prompted us to test whether the identified monosaccharides could modulate SPI-1 expression in *S*. Typhimurium. Specifically, we tested for suppression of the SPI-1 needle protein gene *prgH* in the presence of individual sugars. While the monosaccharide mixture did not suppress SPI-1 expression, D-GalNAc, D-GlcNAc, and, to a lesser extent, D-galactose each suppressed *prgH* (Figure S6A), consistent with the predicted ligand interactions. GalNAc- and GlcNAc-mediated suppression of *prgH* expression was dose-dependent, saturating around 0.2% w/v or approximately 9.0 mM (Figure S6B). A pool of equal parts GalNAc and GlcNAc showed similar results (Figure S6B).

Ligand docking simulations predicted a binding site for GalNAc (Figures 4D and S6C) that contained residues previously implicated in sensing other anti-infective intestinal signals, including bile and short- and long-chain fatty acids.^28,29^ To test whether this same pocket mediates HexNAc signaling, we used a previously characterized “signal-blind” HilD mutant (HilD^Q39E,N44D,H95L^).^29^ This strain failed to downregulate SPI-1 genes in response to GalNAc or GlcNAc (Figure 4E), indicating that these sugars act through this conserved binding pocket. Among the mucin sugars, this effect was specific to the HexNAc monomers, as neither galactose, fucose, nor sialic acid exhibited a similar HilD-dependent effect on SPI-1 gene expression (Figure S6D). Importantly, HilD^Q39E,N44D,H95L^ was also unresponsive to intact MUC2 (Figures S6E and S6F), supporting the role of this domain in mucin sensing. This unresponsiveness was not due to a general loss in HilD function or activity, as evidenced by the high levels of invasion of HT-29 cells (Figure S6E) and WT-level expression of downstream genes in HilD^Q39E,N44D,H95L^ (Figure S6G). Moreover, since the signal-blind mutant retained its high levels of invasion with MUC2 (Figure S6E), the anti-invasion activity of mucin is likely a consequence of its signaling activity rather than a strict barrier or caging effect of the polymer network.

Given that HilD^Q39E,N44D,H95L^ mutants are also resistant to other host-derived anti-infective metabolites,^28,29^ we next tested whether HexNAc interacts with another host signal identified to suppress SPI-1 via the HilD jelly-roll. Co-treatment with either GalNAc or GlcNAc and the bile acid chenodeoxycholic acid (CDCA) revealed an additive suppression of SPI-1 expression (Figure S6H), suggesting combinatorial integration of host cues at the level of HilD. The combined data support the idea that mucin-derived HexNAcs function as host-derived anti-infective signals that suppress SPI-1 expression in *S*. Typhimurium. This suppression appears to occur, at least in part, through additive integration with other host metabolites such as bile acids and may involve direct interaction with the HilD jelly-roll domain. These findings highlight a broader mechanism by which the host mucosal environment modulates bacterial virulence through multiple, converging cues.

### Native presentation of GalNAc on polypeptides potentiates its anti-infective activity

**Figure 5.**
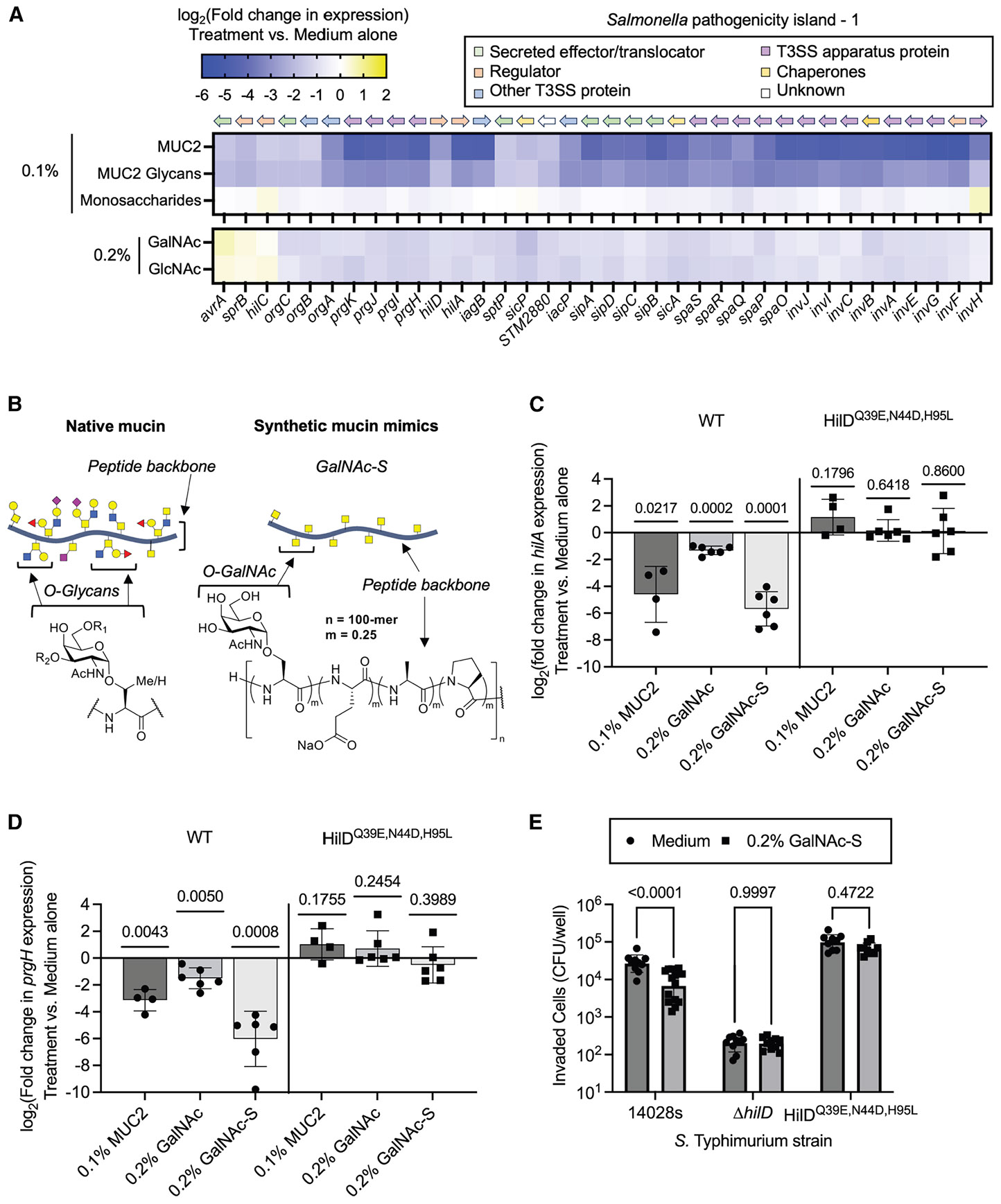
Presenting GalNAc on a peptide backbone promotes its activity (A) Heatmap of SPI-1 genes with genomic orientation and function indicated.^74^ Gene expression changes were determined by RNA sequencing of *Salmonella* cultured with an equal-parts mixture of galactose, GalNAc, GlcNAc, Neu5Ac, and L-fucose (0.1% w/v total monosaccharide, *n* = 2 biologically independent replicates), GalNAc (0.2% w/v, 9.0 mM, *n* = 3), or GlcNAc (0.2% w/v, 9.0 mM, *n* = 3) relative to medium alone on SPI-1 gene expression. MUC2 and MUC2 glycan data (Figures 1E and 2B) are included for comparison. (B) Design of glycopolypeptides using the established N-carboxyanhydride polymerization strategy.^42,43^ (C) Effect of MUC2, GalNAc, or GalNAc-peptides (25% glycan density) on *hilA* gene expression in *S.* Typhimurium 14028s with and without HilD binding site mutations, as measured by qPCR. Bars represent mean log_2_-adjusted changes relative to medium alone. A one-sample t test was performed to evaluate whether the change in expression was significantly different than 0. Exact *p* values are shown above each bar. (D) Effect of MUC2, GalNAc, or GalNAc-peptides (25% glycan density) *prgH* gene expression in *S.* Typhimurium 14028s with and without HilD binding site mutations, as measured by qPCR. Bars represent mean log_2_-adjusted changes relative to medium alone. A one-sample t test was performed to evaluate whether the change in expression was significantly different than 0. Exact *p* values are shown above each bar. (E) Effect of GalNAc-peptides on *S*. Typhimurium host-cell invasion, as determined by enumerating the invaded bacterial cells that survived gentamicin treatment. Bars represent mean log_10_-adjusted CFU/mL. A two-way ANOVA with Šídák’s multiple comparisons test was conducted to evaluate the effect of HilD mutations on GalNAc-peptide-mediated suppression of invasion. Exact *p* values reported. In (C–E), data points represent individual biological replicates, and error bars represent the SD.

To better relate HexNAc-mediated changes in SPI-1 gene expression to mucin- and glycan-mediated changes, we performed RNA-sequencing on LT2 with or without 0.2% (9.0 mM) GalNAc or GlcNAc. Each of these monomer sugars partially inhibited SPI-1; however, the inhibitory effect was less pronounced than for complex mucin glycans or intact MUC2 (Figure 5A). The activity gap between mucins and untethered mucin glycans and the monosaccharides may suggest that the native arrangement of glycans (e.g., presentation on a peptide backbone and/or within a glycan chain) is important for their full activity.

To investigate this, we synthesized GalNAc-serine polypeptides, mimicking the natural presentation of sugars on mucins (Figure 5B). GalNAc-serine polypeptides were designed with a defined length (100-mer) and glycan density (25%) and with spacer residues based on the composition of the native mucin domains.^41-43^ Despite having lower total GalNAc content (~3.2 mM) than provided in the soluble GalNAc treatment (9.0 mM), these glycopolypeptides showed inhibitory activity comparable to intact MUC2, restoring the suppressive effect observed with free GalNAc (Figure 5C: *hilA* expression and 5D: *prgH* expression). Moreover, mutations in the HilD binding site (Q39E, N44D, and H95L)^29^ similarly abolished glycopolypeptide-mediated downregulation of SPI-1 genes (Figures 5C and 5D), indicating that like MUC2 and GalNAc, GalNAc-serine polypeptides signal through HilD. In line with these findings, the GalNAc-serine polypeptides reduced epithelial cell invasion in a HilD-dependent manner (Figure 5E). Thus, mucin-inspired glycopolypeptides, similar to native mucins, exert both transcriptional and phenotypic control over *Salmonella* virulence, highlighting the functional significance of glycan presentation in modulating host-pathogen interactions.

### Sialylation ablates glycopolypeptide anti-SPI-1 activity

**Figure 6.**
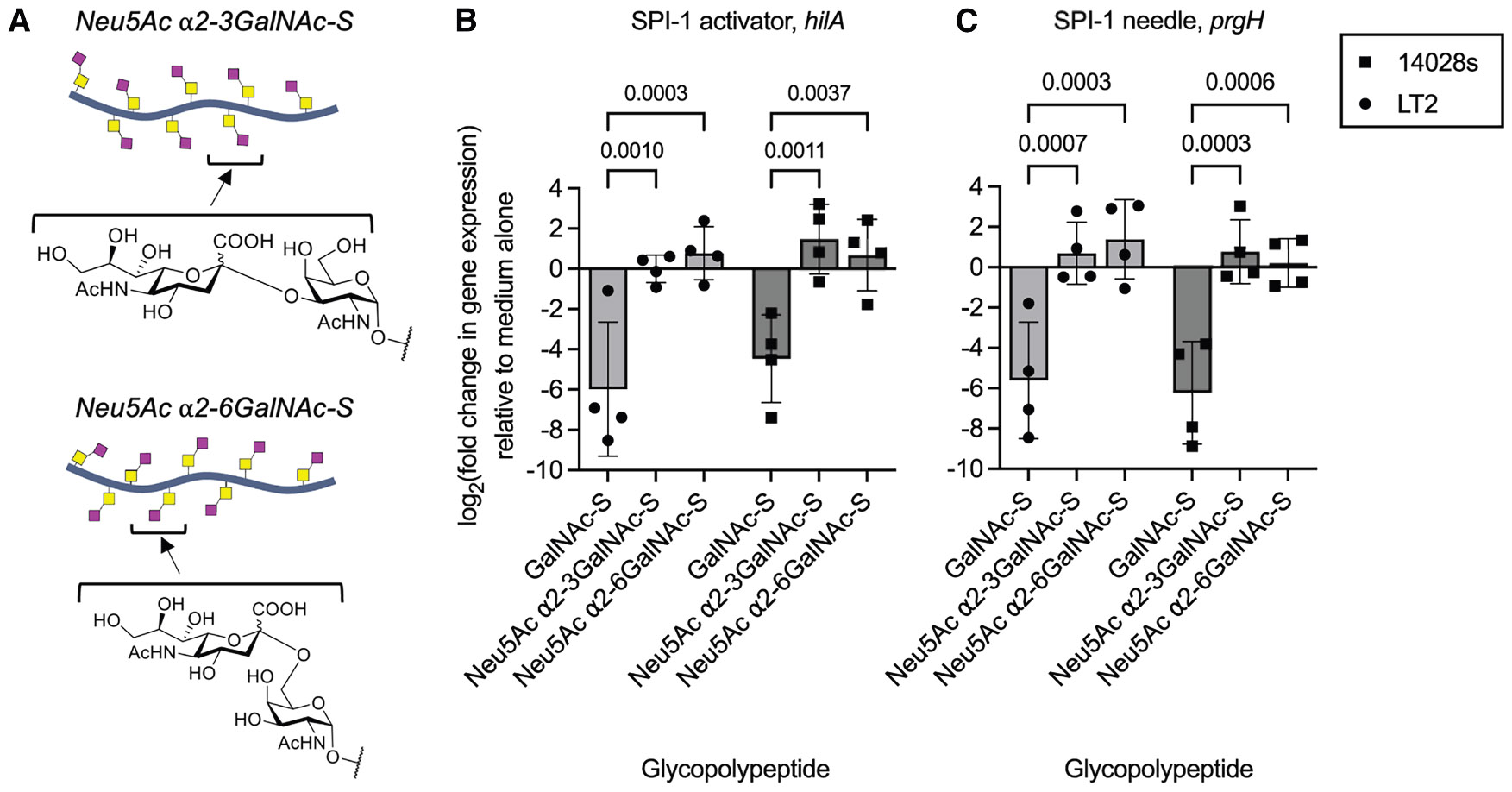
Sialylation ablates glycopolypeptide anti-SPI-1 activity (A) Design of sialylated glycopolypeptides, 100 residues and 25% glycosylation. (B) Effect of sialylated or unsialylated GalNAc-peptides (25% glycan density, 0.1% w/v) on *hilA* gene expression in *S.* Typhimurium LT2 or 14028s, as measured by qPCR. (C) Effect of sialylated or unsialylated GalNAc-peptides (25% glycan density, 0.1% w/v) on *prgH* gene expression in *S.* Typhimurium LT2 or 14028s, as measured by qPCR. In (B) and (C), data points represent individual biological replicates, bars represent mean log_2_-adjusted changes relative to medium alone, and error bars represent the SD. A two-way ANOVA with Dunnett’s multiple comparisons test was conducted to evaluate the effect of sialylation on GalNAc-peptide-mediated suppression of SPI-1 gene expression. Exact *p* values are reported.

Since mucin glycans are frequently capped with terminal sialic acids, we next examined how this common modification influences glycopolypeptide-mediated suppression of *Salmonella* virulence. We hypothesized that terminal sialylation may mask underlying HexNAc residues, thereby interfering with their recognition by bacterial sensors or transporters. To test this, we chemoenzymatically synthesized glycopolypeptides bearing α2,3- or α2,6-linked sialic acids on the GalNAc residues^44^ (Figure 6A). These linkages reflect common terminal structures found on mucins in the gastrointestinal tract.^45-47^ Strikingly, both forms of sialylation fully abolished glycopolypeptide-mediated suppression of SPI-1 genes (*hilA* and *prgH*) in both LT2 and 14028s strains (Figures 6B: *hilA* expression and 6C: *prgH* expression). This suggests that sialylation blocks the interaction of GalNAc-decorated scaffolds with *Salmonella* regulatory machinery, either by preventing uptake, interfering with receptor binding or altering downstream signaling. These results underscore the dual importance of glycan presentation and specific saccharide composition in governing mucin-derived signaling.

Together with our findings on GalNAc-polypeptides and free monosaccharides, this highlights a finely tuned structure-function relationship in mucin glycans, where both accessibility and chemical identity are critical for anti-virulence activity. This insight not only helps to explain how subtle glycosylation changes might influence host-microbe interactions but also informs future efforts to design synthetic glycan scaffolds with targeted anti-infective functions.

## DISCUSSION

Mucus forms a vital barrier against infection, with mouse models highlighting the protective role of intestinal mucin Muc2 and glycans against colitis and pathogens like *Salmonella*.^1,2,48^ Our study uncovers a previously underappreciated mechanism whereby MUC2 inhibits *Salmonella* invasion by suppressing SPI-1 expression via the AraC-type regulator HilD. HilD, while primarily known for its role in virulence regulation, also indirectly influences *Salmonella* metabolism through the metabolic costs of SPI-1 expression.^49,50^ This suggests that mucin glycans may not only impact the virulence potential of *Salmonella* but also its metabolic adaptation to the host environment. While our work focuses on the chemical inhibitory effects of mucin glycans, this inhibition likely acts in concert with the well-established physical barrier functions of mucus. For instance, at high mucus densities, bacterial access to the epithelium may be impeded through steric hindrance and entrapment, while at low densities, glycan-mediated suppression of virulence gene expression may provide an additional level of protection. Together, these complementary mechanisms likely function synergistically to reduce pathogen invasion and maintain mucosal homeostasis.

Consistent with this model of glycan-mediated virulence attenuation, we observed that invasion inhibition and SPI-1 suppression also occurred with soluble mucin glycans and glycopeptides, which lack large structural bulk, indicating that mucin bioactivity is not solely dependent on steric hindrance. Moreover, MUC2 and MUC5AC, but not the anionic mucin analog CMC, showed comparable inhibitory effects, likely reflecting shared motifs within the glycan repertoire.^20,21^ Mutant strains lacking HilD sensing did not exhibit reduced invasion in the presence of mucins, further supporting a signaling-based mechanism.

Although the specific transport mechanisms for mucin and glycans are currently unclear, *S.* Typhimurium was capable of growing on MUC2 as the sole carbon source, indicating its ability to degrade and import mucin components. By deconstructing MUC2, we identified a pool of complex glycans built primarily on structures consistent with the core 1 and core 3 motifs, along with GalNAc and GlcNAc, as inhibitors of SPI-1 gene expression. Notably, while both HexNAc sugars suppress SPI-1 in a HilD-dependent manner, only GlcNAc is utilized as a carbon source,^51^ indicating that glycan utilization is not essential for virulence regulation.

Our results also indicate that the native presentation of mucin sugars on a peptide backbone is important for their activity. Specifically, while HexNAc monosaccharides alone had modest effects on SPI-1 expression, perhaps due to limited avidity and lack of spatial presentation, tethering GalNAc to a peptide backbone enhanced its SPI-1 suppressive activity to levels comparable to native mucins. Moreover, we found that sialylated glycopeptides had no activity in the 14028s or the LT2 strain, suggesting that terminal sialic acid may sterically or electrostatically shield underlying GalNAc residues, thereby preventing signaling in fully sialylated glycopeptides. Interestingly, while sialylation of synthetic glycopeptides in our assays completely abrogated their ability to suppress SPI-1 expression, porcine MUC2, which is partially sialylated, retained inhibitory activity. We hypothesize that the effect is likely driven by the non-sialylated or partially desialylated structures, which represent ca. 70% of the glycan pool. Additionally, *S.* Typhimurium can express multiple putative sialidases, such as NanH and STM1252, that could potentially cleave mucin sialic acids to reveal underlying GalNAc residues.^52,53^ Although our synthetic glycopolypeptides are known substrates for other bacterial sialidases, it is possible that due to the high density capping, sialic acids are less accessible for enzymatic cleavage than those on natural porcine mucin. due to the high density capping. Notably, *S.* Typhimurium LT2 but not 14028s encodes the sialidase, NanH,^54^ that preferentially cleaves the α2,3 linkage.^55^ However, this enzyme is speculated not to have strong activity against sialic acid on mucin or *O*-glycans,^55^ which is consistent with our findings. It will be interesting to explore whether the diverse repertoire of sialidases produced by other gut microbes modulates mucin glycan availability and thereby influences *Salmonella* virulence regulation. Such future studies leveraging selective sialidases will be valuable in exploring how mucin-glycan structure impacts microbial regulation and host interactions.

An important next step will be understanding how glycan activity varies across the gut environment. Various HilD-modulating signals are present at high concentrations throughout the gastrointestinal tract, and their influence on SPI-1 likely shifts with intestinal location and physiological context, reflecting the spatial organization of host secretions, microbial metabolites, and nutrient gradients. For example, bile acids, which are released at high μM to low mM concentrations in the duodenum,^56^ may prime *S*. Typhimurium for reduced SPI-1 activity early in gut transit, while SCFAs produced by the microbiota may play a dominant role in the colon, where they can reach concentrations in the 10–100 mM range.^57^ As with these other gutderived signals, mucin-derived sugars can be estimated to reach mM-range concentrations in the lumen, based on mucin levels of up to 5% w/v and their high glycan content (~70%-80% by mass).^3,58^ Moreover, our data suggest that these intestinal cues may act not only independently but also in combination, as we observed additive suppression of SPI-1 when HexNAc sugars were combined with the bile acid CDCA.

Mucin glycosylation itself shows regional specificity, with increasing sialylation and decreasing fucosylation from the ileum to rectum.^59,60^ Our data suggest that such sialylation may block the anti-SPI-1 activity of mucin glycans, positioning the ileum, where the mucus layer is thicker, less sialylated, and accumulates more rapidly than in the proximal small intestine,^3^ as a potential key site for glycan-mediated *Salmonella* attenuation. Despite the abundance of mucin and other anti-virulence cues in the gut, *S*. Typhimurium remains a highly successful pathogen, likely due to its ability to precisely regulate virulence in response to environmental context. For instance, repression of SPI-1 by host signals such as mucin glycans may help the pathogen avoid premature immune detection during transit through the intestinal lumen, while localized reactivation of SPI-1 where mucus is thinner or disrupted could promote timely invasion. Notably, sialylated MUC1 has been shown to promote *Salmonella* invasion,^61^ highlighting the central role that sialic acid may play in coordinating invasion machinery expression and bacterial colonization strategies. We propose that this spatial layering and potential synergy among anti-virulence signals may tune *S*. Typhimurium invasion potential along the gut axis. Future work should explore how *Salmonella* senses and integrates these mucosal signals to modulate invasion and how this impacts both its virulence and metabolic strategies.

Since HilD is a cytosolic protein, an important unresolved question raised by our findings is whether mucin-derived glycans exert their regulatory effects on bacterial virulence through direct transport into the cell or by acting extracellularly to induce the production of endogenous HilD signals. One possibility is that free glycans, such as GlcNAc and GalNAc, are actively imported by bacterial sugar transporters. For example, *Salmonella* encodes several phosphotransferase systems and other transporters capable of importing monosaccharides, which can influence gut colonization in microbiota-dependent and -independent manners.^62^ This model is consistent with our virtual screening, which suggests these sugars may interact directly with HilD’s carbohydrate-binding domain, similar to how anti-infective bile components and fatty acids signal through HilD.^28,29^ Alternatively, mucin glycans may act as extracellular cues that bind surface sensors, initiating signaling cascades without requiring internalization. In this scenario, glycan multivalency or spatial presentation on mucin backbones could influence receptor clustering or crosslinking, leading to altered signal transduction. Our data do not yet distinguish between these mechanisms. The fact that free and peptide-bound monosaccharides have distinct effects on virulence gene expression suggests that spatial organization and density of glycans presented on the mucin backbone could facilitate stronger or qualitatively different interactions compared to monovalent free sugars. Taken together, these possibilities constitute a working model of how mucin-derived glycans could modulate virulence. Further experiments, such as glycan uptake assays, use of transporter knockouts, or monitoring of intracellular signaling markers, will be necessary to clarify whether the effects are mediated by import and metabolism or by direct surface signaling.

Structurally similar AraC-type virulence regulators are found in other enteric pathogens, including *S. flexneri*, enterotoxigenic *E. coli*, and *V. cholerae*,^35^ suggesting their possible involvement in sensing and responding to the mucosal environment. Indeed, core 2 glycans inhibit *V. cholerae* toxigenic conversion by interfering with the TcpP/ToxR/ToxT virulence pathway.^63^ Notably, ToxT is a HilD homolog, further supporting a possible conserved sensory strategy between these two pathogens. Other work has shown the breadth of anti-infective action of native mucins against diverse pathogens.^21,64-71^ Given the ubiquitous nature of complex sugars in the mucosal environment, as well as in other host glycoproteins, glycolipids, proteoglycans, and human milk oligosaccharides, it is plausible that similar glycan-based signaling mechanisms exist in other microbes to fine-tune behaviors within the host environment. The success of our glycomimetic strategy highlights how decoding the molecular language of mucin glycans can inform the design of precision antivirulence therapeutics inspired by host biology.

### Limitations of the study

This study has several limitations that warrant consideration. Here, we utilized porcine intestinal mucins as a source of *O*-glycans. While porcine mucins share several glycosylation features with human mucins, including all major monosaccharide components, significant differences exist. For example, human MUC2 *O*-glycans possess diverse fucosylation, moderate sialylation, and little sulfation compared to porcine MUC2.^72^ Variations in core glycan structures have also been observed, with one study demonstrating that core 2 and core 4 structures are more abundant in porcine colonic mucus than in human mucins.^73^ Additionally, porcine intestinal mucins are also capped with two forms of sialic acid, Neu5Ac, which is also present in human mucins, and Neu5Gc. These structural differences may influence glycan-mediated interactions with pathogens, and thus, extrapolation of our findings to human systems should be considered within the context of these species-specific differences. Additionally, the release of *O*-glycans via ammonolysis, while effective at generating intact *O*-glycans and scalable enough to facilitate functional studies of mucin-derived glycans, can introduce degradation artifacts such as peeling reactions (for example, the hexose-deoxyhexose structure in Figure 3A). These side reactions may complicate precise structural assignments and could potentially affect the biological activity of the glycans. Future studies employing alternative release methods may mitigate these issues and provide more accurate glycan profiles.

A second limitation is our use of *in silico* docking approaches, which provide hypothetical insights into potential glycan-protein interactions but do not establish direct binding. Moreover, the mechanisms by which mucin-bound or free glycans influence intracellular signaling, whether via direct receptor binding, transport, or induction of endogenous pathways, remain to be elucidated.

Finally, this study relies on *in vitro* tissue culture models, which are valuable for controlled mechanistic investigation but do not capture the full physiological complexity of the gastrointestinal environment. Factors such as the spatial heterogeneity of the mucus layer, the presence of commensal microbiota, and host immune responses will likely influence pathogen behavior. Therefore, animal studies will be critical for assessing the relevance of glycan-based interventions in more dynamic hostmicrobe systems and for dissecting the relative contributions of mucin glycans’ chemical inhibitory functions and the well-established physical barrier properties of mucus.

## STAR★METHODS

### EXPERIMENTAL MODEL AND STUDY PARTICIPANT DETAILS

#### Bacterial strains and growth conditions

This study utilized two strains of *S*. Typhimurium: 14028s and LT2. *S*. Typhimurium mutants were derivatives of S. Typhimurium 14028s. Strain 8640 is a spontaneous, nalidixic acid-resistant isolate of ATCC 14028s.

Overnight cultures of *Salmonella* strains were grown at 37°C, shaking at 220 r.p.m. in liquid Miller lysogeny broth (LB) medium (Becton Dickinson, Difco). The antibiotics chloramphenicol (15 μg/mL), kanamycin (50 μg/mL), carbenicillin (100 μg/mL), and nalidixic acid (64 μg/mL) were used for selection or screening where appropriate.

For gene expression and invasion experiments, overnight *Salmonella* cultures were diluted in SPI-1-inducing LB.3 medium (LB medium with 300 mM NaCl) with or without mucin (MUC2 or MUC5AC), MUC2 glycans, monosaccharides (D-galactose, N-acetyl-D-galactosamine, N-acetyl-D-glucosamine, L-fucose, and/or N-acetylneuraminic acid), or synthetic glycopeptides (GalNAc-S, Neu5Acα2,3-GalNAc-S, or Neu5Acα2,6-GalNAc-S). Bacteria were cultured statically for 3 h in 96-well round-bottom plates.

Note: Commercially purchased N-acetyl-D-galactosamine had variable activity by lot number. We recommend tracking lot numbers for commercially purchased sugars, verifying their identities, and recrystallizing as needed. Lot numbers for the monosaccharides tested in their soluble untethered form are reported in the key resources table.

#### Human HT-29 growth conditions

Human intestinal epithelial HT-29 cells obtained from ATCC were maintained in Dulbecco’s modified eagle medium (DMEM) supplemented with 10% heat-inactivated fetal bovine serum and 1% Penicillin-Streptomycin (P/S) during culture maintenance. Note: Antimicrobials were removed before the invasion assays. Cells were kept at 37°C in a cell culture incubator with 5% CO_2_ and used between passage number 5 and 15. Mycoplasma testing was conducted regularly as part of the cell maintenance practice. Cells were split with Trypsin EDTA when they reached 70% confluency and used for different assays as described in the method details.

#### Mucin isolation from pig tissues

The Institutional Animal Care and Use Committee at Massachusetts Institute of Technology approved the tissue harvest protocol (pig stomachs and intestines) for mucin isolation.

### METHOD DETAILS

#### Generation of *prgH*-GFP fusion strain

The *S*. Typhimurium ATCC 14028s strain 9384 harboring the *gfp*+ gene under transcriptional control of the SPI-1 *prgH* gene promoter (P*prgH-gfp*+) was constructed by standard bacteriophage P22 transduction of strain 8640 using phage lysates prepared on strain JH3010^75^ with selection for chloramphenicol-resistance and screening for GFP expression using fluorescence microscopy.

#### Generation of gene deletion strains

Site-directed, gene deletion/replacement mutagenesis was performed as described by Datsenko and Wanner (2000),^76^ with the exception that the source of the λ Red recombinase was plasmid pSIM6^77^ for deletion of the *fimZ* and *mlc* genes, rather than pKD46, which was used for deletion of the *hilE* gene. Mutagenic PCR products were generated using plasmid pKD4^76^ as the template for amplifying the kanamycin-resistance cassette containing flanking chromosomal homologous sequences for recombination. The primers used to amplify the mutagenic PCR products for deletion of the *fimZ*, *hilE*, and *mlc* genes are listed in Table S2.

Putative kanamycin-resistant, gene deletion mutants were screened by PCR using gene-specific external primers combined with kanamycin cassette internal primers (k1 and k2) to verify the chromosomal location of the gene deletions. After verification, P22 lysates were prepared using the mutant strains as hosts, and the gene deletions were transduced into the wild-type strain (8640) with selection for kanamycin resistance. The chromosomal localization of the gene deletions was again verified using PCR, and the chromosomal kanamycin-resistance cassette was subsequently removed by transformation with the FLP-recombinase expression plasmid, pCP20,^78^ with selection for carbenicillin-resistance at 30°C, followed by screening for loss of kanamycin-resistance. Plasmid pCP20 was subsequently removed by the growth of isolates in liquid culture at 37°C without selection and streaking onto agar plates without selection for growth overnight at 37°C. The following day, cultures of single colonies were used to make glycerol stock cultures and screened for loss of carbenicillin- and chloramphenicol-resistance to verify loss of pCP20.

#### Mucin purification

Native porcine gastric mucin (MUC5AC) was purified from 10 to 20 pig stomachs, and porcine intestinal mucin (MUC2) was purified from 6 to 10 pig small intestines (each of approximately 5–10 m in length) as previously described.^21,63,64,66-68^ Organs and mucus scrapings were kept on ice throughout processing. Stomachs were opened by cutting greater curvature; then excess food was discarded before scraping mucus from the tissue. Intestines were cut into about 30 cm length sections, cut open along the long axis, and scraped to collect mucus. Scrapings were then diluted at a ratio of 1:5 (500 mL scrapings to 2.5 L) using Milli-Q water. NaCl (to 0.2 M), NaN_3_ (to 0.05%), Benzamidine HCl (to 5 mM), dibromoacetophenone (to 1 mM), phenylmethylsulfonylfluoride (PMSF, to 1 mM), and EDTA pH 7 (to 5mM) were added to the diluted scrapings. Scrapings were stirred at 4°C overnight to solubilize mucus. Coarse tissue and food debris were removed by low-speed centrifugation of solubilized mucus at 8000 x g RCF (7,000 rpm Sorvall GS-3 rotor) for 30 min at 4°C. Supernatant was recovered and then centrifuged on an ultracentrifuge at 32,000 x g RCF for 30 min at 15°C (20,000 rpm, Beckman 45 Ti rotor). The central fraction was collected, avoiding the accumulated fats and lipids at the top and the pelleted material at the bottom. Using a Buchner funnel, the supernatant was filtered through 2 wetted Whatman filters. The filtered sample was then concentrated up to 5x, washed twice with 0.2 M NaCl and 0.05% NaN3, and ultracentrifuged for a second time. The collected supernatant (500–800 mL) was then loaded on a Sepharose CL-2B column at 3–5 mL/min. Columns were run and are stored in 0.2 M NaCl, 0.05% NaN_3_. After sample loading, the column was eluted, and 45 mL fractions were collected. Elution peaks were monitored by UV absorbance at 215 nm or 280 nm. Typical 215:280 ratios of the mucin fractions are 5–8:1. Mucin fractions can be confirmed by dot blot. Mucin-containing fractions were collected and pooled, transferred to a Millipore filtration cell containing a pre-wet, prewashed Pall membrane (100,000 MWCO), and concentrated at 4°C. Samples were then iteratively washed with filtered Milli-Q water (until samples were diluted at least 1:1000) to remove salts. Twenty-five mL samples were aliquoted into 50 mL tubes, flash-frozen, and lyophilized. Lyophilized purified mucin was stored at ≤ −20°C until needed. The mucin yield from 10 stomachs or 10 intestines is typically on the order of 100 mg MUC5AC or up to 1 g MUC2. For experiments involving purified mucins, the material was weighed and diluted into the appropriate medium and then solubilized by gentle shaking overnight at 4°C.

#### Mucin glycan isolation

Mucin glycans were isolated from purified MUC2 from porcine intestinal mucus. Glycans were cleaved from the protein mucin backbone via a non-reductive ammonolysis procedure as previously described.^64,69^ A saturated ammonium hydroxide reagent was prepared by mixing 10 mL NH_4_OH with 3 g (NH_4_)_2_CO_3_ and mixed for 3 h in a chemical hood. The ammonia hydroxide reagent was added to the lyophilized mucin to a concentration of 30 mg/mL (e.g., 60 mg mucin in 2 mL ammonia solution). An equal volume of the ammonia solution was added to an empty tube to serve as a blank control. The mucin-ammonia mixture was then incubated in a 50°C sand bath for 60 h. Every 12 h, 0.1 mg (NH_4_)_2_CO_3_ was added per 1 mL sample and vigorously vortexed. The mucin-ammonium mix was then dried by centrifugal evaporation in a SpeedVac with a vapor trap. Dried samples were iteratively washed by resuspending the sample in H_2_O and drying by centrifugal evaporation until all (NH_4_)_2_CO_3_ was removed from the blank. These glycosylamines were then converted to free, reducing glycans by adding 1.0 mL of 0.5M boric acid to each sample and incubating at 37°C for 1 h. Glycans were then dried by centrifugal evaporation and then iteratively washed by resuspending the sample in methanol and drying by centrifugal evaporation until the blank control was empty. Glycans were then separated from the peptide backbone by spin dialysis. Specifically, all samples were dissolved in HPLC-grade water and centrifuged at 4°C against a pre-washed Amicon 10k MWCO membrane. The concentration of the glycan solution was then quantified using a Phenol/Sulfuric Assay and a glucose standard curve ranging from 0 to 10 mg/mL glucose. For the Phenol/Sulfuric Assay, 10 μL of standards or sample was added to the wells of a 96-well plate and mixed with 100 μL of concentrated sulfuric acid and then 30 μL of 5% phenol. The plate was incubated at room temperature for 15 min to allow the color to develop, and then the absorbance was measured at 420 nm on a plate reader. The weight percentage of the glycans was calculated based on the linear relationship of the glucose concentration to absorbance. Samples were then aliquoted to the desired mass per tube, dried on the SpeedVac, and stored at −20°C.

#### Glycan analysis by LC-NSI-MS

Released O-glycans were permethylated by using methyl iodide on DMSO/NaOH mixture. Briefly, the dried material was dissolved and transferred to a glass tube in dimethyl sulfoxide and methylated by using methyl iodide on DMSO/NaOH slurry mixture. The reaction was quenched with water and the reaction mixture was extracted with dichloromethane and dried. Dried, permethylated O-glycans were re-dissolved in a solution of 50μL methanol then an aliquot was taken for LC-MS. Samples were then run on a Themo Orbitrap Fusion Tribrid coupled to a Thermo Ultimate3000 RSLC nano chromatography system. Following injection, analytes were separated on a commercial C18 nano column before being directed into the mass spectrometer. A top-down automated MS/MS program collected full MS spectra as well as MS/MS (CID fragmentation) of the eluent over a 60-min profile. The resulting data collected was hand-annotated based on compositional total mass as well as MS/MS, assisted by GlycoworkBench and Glycomod, by the Analytical Service & Training Laboratory at the Complex Carbohydrates Research Center, University of Georgia.

#### Glycopolypeptide preparation, chemoenzymatic synthesis

αGalNAc(OAc)_3_Ser,^41^ Pro,^79^ tBuGlu,^41^ and Ala^41^ *N*-carboxyanhydrides (NCAs) were prepared according to literature procedures. All NCA polymerizations were performed in an N_2_-filled glovebox. NCAs were suspended at 50 mg/mL in anhydrous THF and combined at a molar ratio of 1:1:1:1 of each NCA. Polymerizations were initiated via rapid addition of 30 mg/mL in dry THF of (PMe_3_)_4_Co catalyst at a monomer to initiator ratio of 30:1. Polymerizations were heated at 50°C in a glovebox for 16 h. Reaction progress was monitored via ATR-FTIR on a Bruker Alpha Spectrophotometer. Upon reaction completion, polymers were analyzed via tandem gel permeation chromatography/light scattering (GPC/LS) with an Agilent 1260 Infinity liquid chromatography pump equipped with Wyatt DAWN HELEOS-II light scattering (LS) and Wyatt Optilab T-rEX refractive index (RI) detectors. Glycopolypeptides were passed through 10^5^, 10^4^, and 10^3^ Å Phenominex Phenogel 5 μm columns in an eluent of 0.1 M LiBr in DMF at 60°°C at a concentration of 3 mg/mL. Glycopolypeptide chemical protecting groups were removed according to literature protocols.^80^

GalNAc groups were sialylated using a one-pot multi-enzyme procedure.^44^ In brief, glycopolypeptides were treated with a sialic acid aldolase (GenBank WP_000224714), a CMP-sialic acid synthetase (GenBank WP_002215295), and either a a2.3 sialyltransferase (GenBank NC_002663) or a a2.6 sialyltransferase (GenBank AAK02272) with ManNAc, sodium pyruvate, and CTP in 100 mM Tris-HCl pH 7.5 with MgCl_2_. Sialylations were allowed to proceed overnight at ambient temperature with gentle agitation at 100 RPM. Polyhistidine-tagged enzymes were removed via Ni-NTA magnetic affinity beads and the resulting sialylated glycopolypeptides were dialyzed against ultrapure water in 2 kDa tubing 4 times every 4–24 h. Purified products were sterile filtered through a 0.22 mm filter and lyophilized to yield fluffy white powders.

#### Cryogenic scanning electron microscopy

Overnight *Salmonella* cultures were washed with SPI-1-inducing LB.3 medium and then resuspended in LB.3 medium with 0.4% (w/v) MUC2. Samples were not washed prior to SEM preparation, as our goal was to visualize the general architecture of the mucin matrix and the apparent embedding of bacteria, rather than to distinguish bound versus unbound bacterial populations. Samples were plunge-frozen by immersion in liquid ethane, which was cooled using a bath of liquid nitrogen. These frozen samples were then stored in liquid nitrogen until further processing. For fracturing and coating, the samples were transferred into a Leica EM VCT500 vacuum transfer system and moved to a Leica ACE9000 freeze fracture system. Each sample was fractured and then etched for 20 min. Subsequently, the samples were coated with platinum and carbon using the Leica ACE9000 ebeam coater. The coated samples were again transferred using the Leica EM VCT500 vacuum transfer system to a Zeiss Crossbeam 540 SEM/FIB. Throughout this process, the samples were maintained under constant vacuum to prevent thawing and ensure they remained at low temperatures. Images were acquired using the Zeiss Crossbeam 540 SEM/FIB at an accelerating voltage of 2 kV, employing both the InLens and secondary electron secondary ions SESI detectors. The acquired images were analyzed using ImageJ software (NIH Bethesda) and were false-colored using Adobe Photoshop for enhanced visualization.

#### Invasion assay

HT-29 cells (human colon epithelial cell line; ATCC, HTB-38) were seeded in 96-well tissue culture plates (2.5 x 10^5^ cells/well) and grown for 18-24 h to ~90% confluency. For invasion assays of polarized epithelial cells, HT-29 cells were seeded at 1 × 10^5^ cells/well onto polyester Transwell inserts (Corning, 0.4 μm pore size, 6.5 mm diameter) placed into 24-well plates as detailed in.^81^ The apical chamber received 200 μL of DMEM without antibiotics, while the basolateral chamber received 500 μL. The apical and basolateral medium was replaced every 48 h, taking care not to disturb the cell monolayer. Any air bubbles beneath the inserts were eliminated, as trapped bubbles can interfere with monolayer integrity. Cells were cultured on inserts for 7 days to achieve a confluent, polarized monolayer.

On the day of the invasion assay, dilutions (1:50) of overnight LB cultures of *S*. Typhimurium strain LT2 or 14028s (WT or mutant) were grown statically in 100 μL of SPI-1-inducing LB (300 mM NaCl, with or without mucin) for 3 h at 37°C. *Salmonella* cells were added to wells at a multiplicity of infection (MOI) of 20:1. Cells were incubated at 37°C and 5% CO_2_ for 2 h to allow invasion. The medium was removed, and the total number of extracellular bacteria was determined by serial dilution and drop-spotting on LB agar plates. HT-29 cells were washed three times with DMEM containing 100 μg/mL gentamicin. Fresh DMEM containing 100 μg/mL gentamicin was added, and cells were incubated for 1 h to kill extracellular *Salmonella*. Wells were then washed three times with fresh DMEM, and cells were lysed with 50 μL of 1% Triton X-100 in 0.9% NaCl for 15 min. Lysates were serially diluted and drop-spotted on LB agar plates to determine the number of invaded bacteria. Data are presented as CFU/well and as log_2_((invaded CFU/total CFU)_treated_/(invaded CFU/total CFU)_control_) to normalize for variability in inocula across experiments and more clearly depict changes in invasion.

#### Confocal imaging of invasion

Dilutions (1:50) of overnight LB cultures of *S.* Typhimurium strain 14028s were grown statically in 100 μL of SPI-1-inducing LB (with or without MUC2) for 3 h at 37°C. HT-29 cells were seeded in a 96-well glass bottom plate (2.5 x 10^5^ cells/well). *Salmonella* cells were then stained with Syto63 nucleic acid stain and added to wells at a multiplicity of infection (MOI) of 200:1. Cells were incubated at 37°C and 5% CO2 for 2 h to allow invasion. HT-29 cells were washed three times with DMEM containing 100 μg/mL gentamicin. Fresh DMEM containing 100 μg/mL gentamicin was added, and cells were incubated for 1 h to kill extracellular *Salmonella*. Wells were then washed three times with fresh DMEM, stained with DAPI and Live/Dead dye, and incubated for an additional 30 min. Images were acquired approximately 4 h after the initiation of the invasion assay with a confocal laser-scanning microscope (LSM 800; Zeiss) with an x63/1.4 NA oil-immersion objective. Images were analyzed with the Zeiss Zen v2.1 and Imaris 9.3.0 imaging software.

##### RNA extraction

*S.* Typhimurium LT2 or 14028s overnight cultures were diluted 1:100 in 100 μL of SPI-1-inducing LB.3 medium (with or without 0.1% MUC2, 0.1% MUC2 glycans, 0.2% GalNAc, 0.2% GlcNAc, 0.1% monosaccharide pool containing equal volumes of D-galactose, D-GalNAc, D-GlcNAc, L-fucose, and Neu5AC), or glycopolypeptides for 3 h at 37°C, under static conditions. RNA was extracted with the MasterPure RNA Purification Kit (Biosearch Technologies), and DNA was eliminated using the Turbo DNA-Free Kit (Ambion).

#### RNA-sequencing

The Agilent 2100 Bioanalyzer (Agilent Technologies) was used to assess RNA integrity. rRNA was depleted with the NEBNext rRNA Depletion Kit for bacteria (NEB). The Illumina HiSeq platform was used for the sequencing mucin-, glycan-treated, and monosaccharide pool-treated samples with a single-end protocol and read lengths of 40 nucleotides. The Illumina NovaSeq platform was used for sequencing HexNAc-treated samples with a paired-end protocol and read lengths of 50 nucleotides. Reads were analyzed on the Galaxy platform^82^ with the Burrows-Wheeler algorithm,^83^ HTseq-count,^84^ and DESeq2.^85^ For all analyses of the sequencing data, we considered FDR-adjusted *p* values <0.05 as significant.

Venn diagrams and overlapping gene counts were calculated in R using VennDiagram,^86^ and KEGG pathway enrichment in the up and downregulated gene sets (∣log_2_(foldchange)∣ > 1 and FDR-adjusted *p* values <0.05) were determined using the enrichKEGG function of the clusterProfiler package.^87^

#### Quantitative PCR

Reverse transcription was performed by first-strand cDNA synthesis from RNA (ProtoScript II First Strand cDNA Synthesis kit, NEB), which was then used as a template for RT-qPCR with the SYBR PowerUp Master Mix (Applied Biosystems) on a Roche LightCycler 480 Real-time PCR system. The primers used in this study are listed in Table S3. The gene *gyrB* was used as a control. Gene expression changes were calculated based on mean change in qPCR cycle threshold (ΔCt) with the ΔΔCt method (fold change = 2^−ΔΔCt^).

#### Growth time course

Overnight cultures of *S.* Typhimurium LT2 were grown in LB media and were diluted to 1:100 in 100 μL LB.3 media ± MUC2 were incubated at 37°C. Growth was evaluated at 0, 2, 4, 8, and 24 h by plating serial dilutions of CFUs onto LB agar plates.

Overnight cultures of *S.* Typhimurium 14028s were diluted to 1:100 in 100 μL M9 media with 0.1% glucose or 0.1% MUC2 as the sole carbon source. Plates were incubated at 37°C, and growth was evaluated by measuring optical density at 0, 2, 4, 6, 8, and 24 h.

#### Data-independent acquisition (DIA)-based quantitative proteomic analysis

*S.* Typhimurium LT2 overnight cultures were diluted 1:100 in 300 μL of SPI-1-inducing LB.3 medium (with or without 0.1% MUC2) for 3 h at 37°C, under static conditions. Cultures were collected and washed two times with water. Bacterial pellets were then resuspend in 200 μL of 1X Bacterial Protein Extraction Reagent (Thermo) with 2 μL of 100X Halt Protease Inhibitor Cocktail (Invitrogen). Cells were Incubated for 15 min at room temperature, then isoluble proteins were precipitated by centrifugation (13,000 xg for 5 min). Protein content in the cell lysate was quantified using the Pierce BCA Protein Assay Kit (Thermo).

Quantitative proteomic profiling using DIA was performed by MtoZ Biolabs. Protein samples (50 μg) were prepared in 100 μL of 50 mM ammonium bicarbonate (NH_4_HCO_3_) containing 10 mM dithiothreitol (DTT), followed by incubation at 56°C for 1 h. Iodoacetamide (IAM) was then added to a final concentration of 20 mM and incubated in the dark at room temperature for 1 h. Residual IAM was quenched by adding DTT to a final concentration of 10 mM, followed by an additional incubation at 56°C for 1 h. Proteins were digested using single-pot solid-phase-enhanced sample preparation (SP3) with a 1:1 mixture of magnetic beads. Beads were washed three times with ultrapure water, then resuspended to 100 μg/μL in water and stored at 4°C. For digestion, 10 μL of the bead suspension was added to 100 μg of protein sample, followed by 110 μL of absolute ethanol. Samples were incubated at room temperature for 15 min to allow protein binding. Beads were separated on a magnetic rack, and the supernatant (binding SQ) was collected and stored at −80°C. Beads were washed three times with 500 μL of 80% ethanol, then air-dried. Proteins bound to the beads were resuspended in 300 μL of 50 mM NH_4_HCO_3_ and digested with 2 μg of trypsin (0.25 μg/μL) at 37°C for 14–18 h with gentle agitation (1000 rpm). Digested peptides were separated from the beads using a magnetic rack, and the clarified supernatant was collected. Samples were lyophilized, and peptides were desalted using a C18 column. Eluted peptides were subsequently dried in a vacuum centrifugal concentrator at 45°C prior to downstream analysis, and resuspended in 0.1% formic acid prior to mass spectrometry analysis.

Peptides were separated on a 5.5 cm High Throughput μPAC *Neo* HPLC column at a flow rate of 250 μL/min. The mobile phases consisted of 0.1% formic acid in water (A) and 80% acetonitrile with 0.1% formic acid (B). The gradient conditions were as follows: 4% B at 0 min, 20% B at 4.0 min, 35% B at 5.8 min, 99% B at 6.2 min, and held at 99% B until 6.9 min. Each run lasted 6.9 min.

DIA mass spectrometry was performed with a full scan range of m/z 380–980. First-stage MS resolution was set to 240,000, with an AGC target of 500% and maximum injection time (IT) of 5 ms. For MS/MS acquisition, AGC was set to 500% and maximum IT to 3 ms. Peptide fragmentation was carried out with a collision energy of 25%. Raw mass spectrometry data (.raw files) were generated for downstream analysis. Peptide and protein identification was performed using DIA-NN (v1.9). The search parameters were as follows: Fixed modification: Carbamidomethylation (C), Variable modifications: Oxidation (M), Acetylation (peptide N terminus), Enzyme: Trypsin, Database: *Salmonella enterica* UP000001014_99287.fasta (UniProt), Maximum missed cleavages: 2, Peptide mass tolerance: 20 ppm, Fragment mass tolerance: 20 ppm.

#### Analysis of *prgH* promoter activity in *Salmonella*

An *S.* Typhimurium strain 9384 overnight culture was diluted 1:100 in SPI-1-inducing LB.3 medium with or without individual or mixtures of monosaccharides (100 μL cultures in a 96-well plate). After 6 h of growth at 37°C, growth was evaluated by measuring the absorbance at 600 nm, and GFP fluorescent intensity was measured using an excitation wavelength of 485 nm and emissions wavelength of 528 nm. Fluorescence was normalized to the optical density and then compared to the fluorescence of *S.* Typhimurium cultured in medium alone.

#### Protein structure modeling and quality assessment

Three protein structure prediction programs were employed to generate 3D models of HilD: trRosetta,^33^ AlphaFold2,^88^ and ESM.^89^ The quality and consistency of the predicted structures were evaluated using QMEAN (Qualitative Model Energy Analysis).^90^ QMEAN utilizes a statistical potential to assess various structural features, including non-bonded interactions, torsion angles, and solvent accessibility. It provides a composite score reflecting the overall quality of each model. Both QMEAN scores and Root-Mean-Square Deviation (RMSD calculations performed in PyMOL (Molecular Graphics System, Version 2.0, Schrödinger, LLC.) were used to compare the models. All predicted structures were very similar; docking studies were performed with the trRosetta model.

#### Pocket identification, docking, and analysis

PocketMiner^34^ software was used to identify potential binding pockets on the predicted HilD structure based on size, shape, hydrophobicity, and potential hydrogen-bonding interactions. A local installation of AutoDock Vina^36,37^ was employed to virtually screen monosaccharides for potential binding. Three-dimensional monosaccharide models (for GalNAc, GlcNAc, Galactose, Fucose, and Mannose) were prepared using GlyCam.^91^ Protein and ligand preparation for docking was performed using AutoDock Tools.^92^ All molecules were converted into the PDBQT format required by AutoDock Vina. For ligand and receptor preparation, hydrogen atoms were added to reflect a physiological pH of 7.4. The search space (20 × 20 × 20 Å) was centered on the predicted binding pocket, and the exhaustiveness parameter was set to the default 32 for thorough exploration. Twenty poses were generated for each ligand to account for conformational flexibility. Docking poses were analyzed based on binding affinity (ΔG) from AutoDock Vina, with lower ΔG indicating stronger binding. Visual inspection using PyMOL (Molecular Graphics System, Version 2.0 Schrödinger, LLC.) and Chimera^93^ software assessed specific interactions between ligands and binding pocket residues.

### QUANTIFICATION AND STATISTICAL ANALYSIS

Statistical data analysis was performed using GraphPad PRISM 10. Fold changes in invasion and gene expression levels were transformed logarithmically before statistical analysis. All statistical details of the experiments, including the statistical tests used, the exact value of n, what n represents, the definition of center, and dispersion and precision measures, can be found in the figure legends. Exact *p* values were reported, and significance was *p* < 0.05.

## Supplementary Material

Supplemental Material Supplemental information can be found online at https://doi.org/10.1016/j.celrep.2025.116304.

## Figures and Tables

**Figurte 1.**
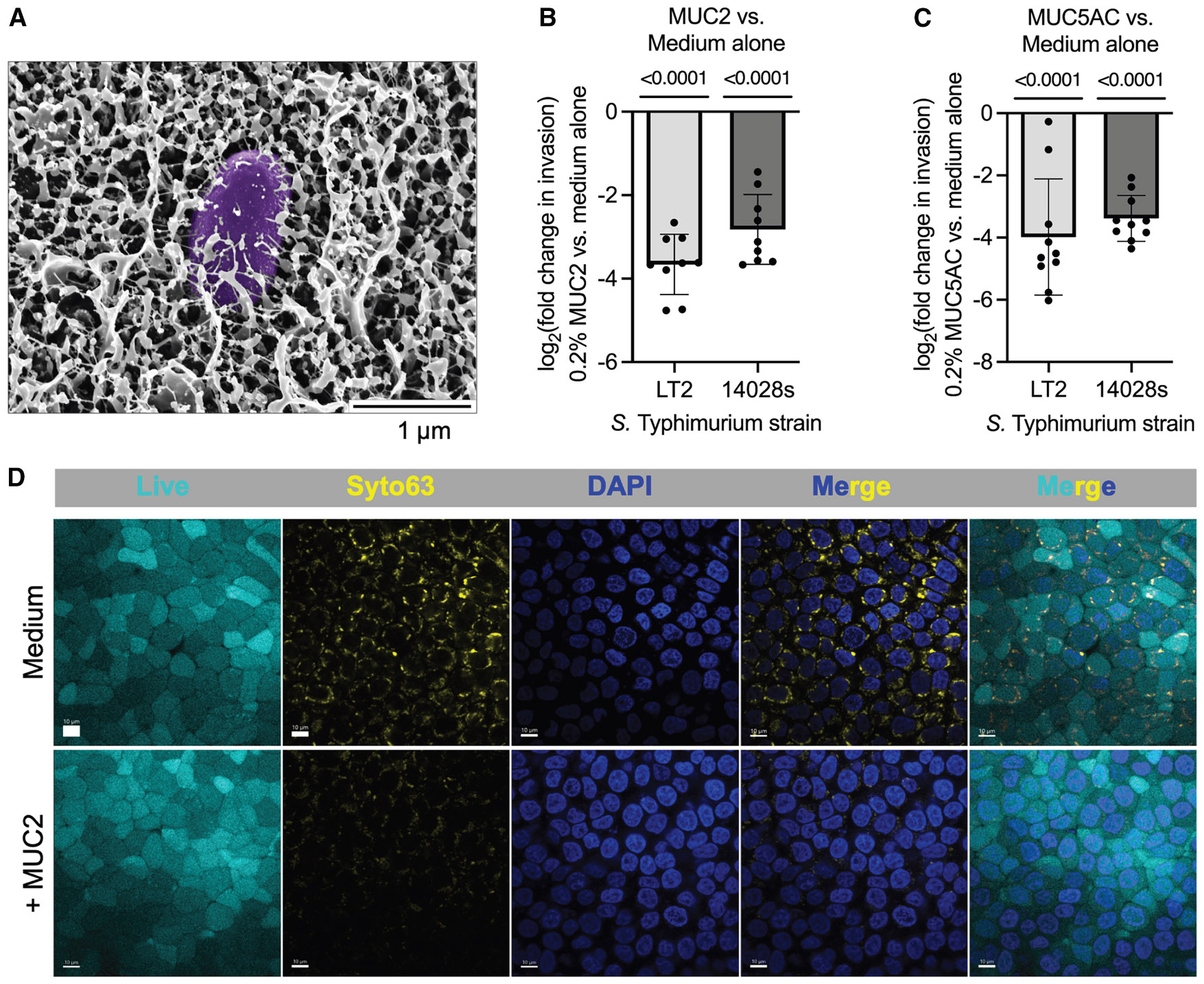
Gastrointestinal mucins inhibit host cell invasion by *S.* Typhimurium (A) Scanning electron microscopy image of *S*. Typhimurium (false-colored in purple) grown with purified MUC2 (0.4% w/v) illustrates the mucin matrix structure and the apparent presence of embedded bacteria. Scale bar, 1 μm. (B) Host-cell invasion, as determined by a gentamicin protection assay of *S*. Typhimurium grown with purified porcine MUC2 (0.2% w/v), of HT-29 cells infected at a multiplicity of infection (MOI) of 20. A one-sample t test was performed to evaluate whether the change in invasion was significantly different than 0. Exact *p* values are shown above each bar. (C) Host-cell invasion, as determined by a gentamicin protection assay of *S*. Typhimurium grown with purified MUC5AC (0.2% w/v), of HT-29 cells infected at an MOI of 20. A one-sample t test was performed to evaluate whether the change in invasion was significantly different than 0. Exact *p* values are shown above each bar. (D) Confocal image of *Salmonella* invasion in medium with or without purified MUC2. Gentamicin protection assay of *S*. Typhimurium grown with MUC2 (0.2% w/v) infecting HT-29 cells at an MOI of 200. Scale bars, 10 μm. In (B) and (C), data points represent individual biological replicates, bars represent mean log_2_-adjusted changes relative to medium alone, and error bars represent the SD. See also Figures S1 and ^S3^.

**Figurte 2:**
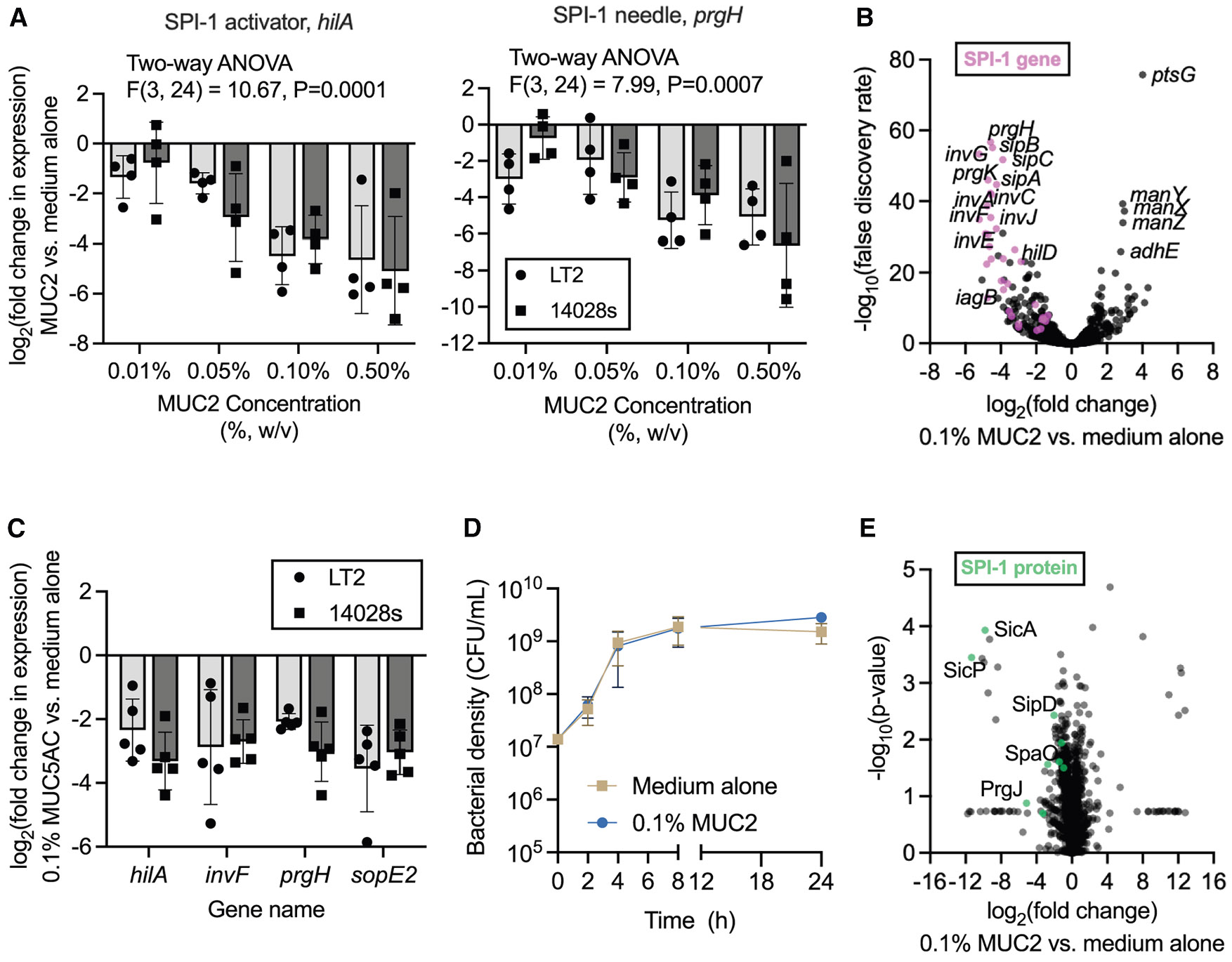
Gastrointestinal mucins downregulate the *Salmonella* pathogenicity island SPI-1 (A) Effect of MUC2 on SPI-1 gene expression, as measured by qPCR. A two-way ANOVA was conducted to evaluate the effect of MUC2 concentration and *Salmonella* strain on SPI-1 gene expression. There was a significant effect of MUC2 concentration on SPI-1 expression. Exact *p* values reported. (B) Gene expression changes, as determined by RNA sequencing, of *Salmonella* LT2 cultured with MUC2 (0.1% w/v) relative to medium alone (*n* = 3 biologically independent replicates). SPI-1 genes are denoted in pink. (C) Effect of MUC5AC (0.1% w/v) on SPI-1 gene expression, as measured by qPCR. (D) *Salmonella* LT2 growth in the presence or absence of MUC2 (0.1% w/v). Data points represent the median colony-forming unit (CFU)/mL (*n* = 3 biologically independent replicates), and error bars represent the 95% confidence interval. (E) Proteomic changes, as determined by DIA proteomic analysis, of *Salmonella* LT2 cultured with MUC2 (0.1% w/v) relative to medium alone (*n* = 3 biologically independent replicates). SPI-1 proteins are denoted in green. In (A) and (C), data points represent individual biological replicates, bars represent mean log_2_-adjusted changes relative to medium alone, and error bars represent the SD. See also Figures S2 and ^S3^.

**Table:** KEY RESOURCES TABLE

REAGENT or RESOURCE	SOURCE	IDENTIFIER
Bacterial and virus strains		
*Salmonella enterica* serovar Typhimurium LT2	ATCC	700720
*Salmonella enterica* serovar Typhimurium ATCC 14028s *gyrA*(D87Y)	M. Fulde	8640
*Salmonella enterica* serovar Typhimurium ATCC 14028s *gyrA*(D87Y) *putP*::P*prgH*-*gfp*+:*cat*::*putA*	K. Tedin	9384
*Salmonella enterica* serovar Typhimurium ATCC 14028s *gyrA*(D87Y) Δ*mlc*	K. Tedin	12294
*Salmonella enterica* serovar Typhimurium ATCC 14028s *gyrA*(D87Y) Δ*hilE*	K. Tedin	12314
*Salmonella enterica* serovar Typhimurium ATCC 14028s *gyrA*(D87Y) Δ*fimZ*	K. Tedin	12346
*Salmonella enterica* serovar Typhimurium ATCC 14028s HilA-HA	H. Hang;^94^	N/A
*Salmonella enterica* serovar Typhimurium ATCC 14028s HilA-HA, *ΔhilD*	H. Hang;^29^	N/A
*Salmonella enterica* serovar Typhimurium ATCC 14028s HilA-HA, HilD^Q39E,N44D,H95L^	H. Hang;^29^	N/A
*Salmonella enterica* serovar Typhimurium SL1344 P*prgH–gfp* JH3010	J.C.D. Hinton;^75^	N/A
TOP10 cells	Thermo Fisher	Cat #C404010
BL21 cells	Thermo Fisher	Cat #C600003
Biological samples		
Porcine Intestines	Research 87, Boylston, MA 01505	N/A
Porcine Stomachs	LeMay and Sons Beef Packaging, Goffstown, NH 03045	N/A
Chemicals, peptides, and recombinant proteins		
LB Broth, Miller (Luria Bertani)	BD Difco	Cat# 244620
Agar	Sigma	Cat# A1296
M9	AMRESCO	Cat# J863-500G
Dextrose	Sigma	Cat# G6152-1KG Lot# SLCB6282
NaCl	Sigma	Cat# S9888-5KG
MgSO_4_	Sigma	Cat# M2773-500G
CaCl	Sigma	Cat# C5670-100G
Chloramphenicol	MPBio	Cat# 190321
Kanamycin	Sigma	Cat# K1377
Carbenicillin	Sigma	Cat# C3416-250MG
Nalidixic acid	Sigma	Cat# N4382
Dulbecco’s Minimum Essential Media (DMEM)	Gibco	Cat# 11965
Fetal Bovine Serum (FBS)	Gibco	Cat# 10500064
DPBS	Gibco	Cat# 14040133
Gentamicin	Sigma	Cat# G1264
Triton X-100	Sigma	Cat# T9284-500ML
NaN_3_	Sigma	Cat# S2002-100G
NH_4_OH, Solution 30–33% NH_3_ in H_2_O	Sigma	Cat# 05002-1L
(NH_4_)_2_CO_3_	Sigma	Cat# 207861-500G
Boric Acid	Sigma	Cat# B6768
Benzamidine HCl	Sigma	Cat# 434760-25G
EDTA	Sigma	Cat# EDS-500G
2,3^′^-Dibromoacetophenone	Sigma	Cat# 654787-5G
Phenylmethylsulphonyl Fluoride	Sigma	Cat# 78830-5G
D-Galactose	Sigma	Cat# G0625-100G Lot# 060M00631V
N-acetyl-D-galactosamine	Sigma	Cat# A2795-500MG Lot# BCCD6801
N-acetyl-D-glucosamine	Fluka	Cat# WA20549 Lot# 1323294
L-Fucose	Sigma	Cat# A16789.03 Lot# R08J007
N-Acetylneuraminic acid	Sigma	Cat# A0812-100MG Lot# BCCD5508
LB broth (glycopolypeptide synthesis)	Invitrogen	Cat# 12780-052
Terrific broth (glycopolypeptide synthesis)	Invitrogen	Cat# 22711-022
LB agar (glycopolypeptide synthesis)	Invitrogen	Cat# 22700-025
Carbenicillin (glycopolypeptide synthesis)	Thermo Fisher	Cat# J61949-06
Imidazole (glycopolypeptide synthesis)	VWR	Cat# 0527-100G
Tris (glycopolypeptide synthesis)	VWR	Cat# 0497-500G
NaCl (glycopolypeptide synthesis)	Fisher	Cat# S271-3
Glycerol (glycopolypeptide synthesis)	Fisher	Cat# BP229-4
CTP (glycopolypeptide synthesis)	Alfa Aesar	Cat# J62238
Sodium pyruvate (glycopolypeptide synthesis)	VWR	Cat# 0342-100G
ManNAc (glycopolypeptide synthesis)	Alfa Aesar	Cat# L11167
MgCl^2^ (glycopolypeptide synthesis)	Fisher	Cat# BP214-500
Critical commercial assays		
MasterPure Complete DNA and RNA Purification Kit	Biosearch Technologies	Cat# MC85200 Lot# 34146
ProtoScript II First Strand cDNA Synthesis kit	NEB	Cat# E6560L
Turbo DNA-Free Kit	Invitrogen	Cat# AM1907
SYBR PowerUp Master Mix	Applied Biosystems	Cat# A25778
NEBNext rRNA Depletion Kit	NEB	Cat# E7850X
LIVE/DEAD Cell Imaging Kit	Invitrogen	Cat# R37601
B-PER Reagent	Thermo scientific	Cat# 78243
Halt Protease Inhibitor Cocktail (100X)	Invitrogen	Cat# 87786
Pierce BCA Protein Assay Kit	Thermo scientific	Cat# 23227
Deposited data		
RNA-seq	This study	GEO: GSE278090
Underlying numerical data for main figures	This study	Dryad: https://doi.org/10.5061/dryad.2bvq83c38
Experimental models: Cell lines		
HT-29	ATCC	HTB-38
Oligonucleotides		
See Table S2 for PCR primer	This Study	N/A
See Table S3 for qPCR primers	This Study	N/A
Recombinant DNA		
Plasmid: pCP20	Cherepanov and Wackernagel^78^	N/A
Plasmid: pKD4	Datsenko and Wanner^76^	N/A
Plasmid: pSIM5	Sharan et al.^77^	N/A
Software and algorithms		
Galaxy version 24.1.0	Galaxy Project	https://docs.galaxyproject.org
Prism 10.3.0	GraphPad	https://www.graphpad.com
R Studio Version 2022.12.0 + 353	Rstudio	https://www.rstudio.com
Zen v2.1	Zeiss	https://www.zeiss.com/microscopy/en/products/software
Imaris 9.3.0	Oxford Instruments	https://imaris.oxinst.com/
PyMOL Version 2.0	Molecular Graphics System, Schrödinger, LLC.	https://www.pymol.org/
AutoDock Vina	Center for Computational Structural Biology at The Scripps Research Institute^36,37^	https://vina.scripps.edu/
PocketMiner	Meller et al.^34^	https://pocketminer.azurewebsites.net/
GlyCam	Complex Carbohydrate Research Center at the University of Georgia^91^	https://glycam.org/
QMEAN	Swiss-model^90^	https://swissmodel.expasy.org/qmean/
trRosetta	Du et al.^33^	https://yanglab.qd.sdu.edu.cn/trRosetta/
AlphaFold2	Jumper et al.^88^	https://alphafold.ebi.ac.uk/
Chimera	UCSF Chimera^93^	https://www.cgl.ucsf.edu/chimera/download.html
Other		
Whatman3 filters	Sigma	#WHA1003150
Amicon Stirred Cell 400 mL	Millipore	MPUFSC40001
Omega 100k membranes	Pall, Fisher Scientific	OM100076
NanoDrop One	Thermo Scientific	ND-ONE-W
Amicon Ultra 10k MWCO centrifugal filters	Millipore	UFC9010
Savant Refrigerated Vapor Trap	Thermo Scientific	RVT5105
Speedvac Concentrator	Savant	SVC100H
Microplate reader	Agilent	BioTek Synergy H1
Ultracentrifuge	Beckman	L8-70M
PCR Microplate	Axygen	PCR-384-LC480WNFBC
96-well tissue culture plates, nucleon Nunc Edge	Thermo Scientific	Cat# 167425
96-well plates, cell star greiner bio-one	Greiner	Cat# 650185
Nickel columns	Thermo Fisher	Cat# 88221
Corning Transwells, PET membrane, 6.5 mm, 0.4μm pore size	Sigma	Cat# CLS3470-48EA

## Highlights

- Intestinal mucin (MUC2) inhibits *S.* Typhimurium invasion by suppressing SPI-1 expression
- MUC2 acts through HilD, a master regulator of the SPI-1 encoded type 3 secretion system
- Mucin-derived sugars are sufficient to downregulate SPI-1
- Synthetic glycopolypeptides recapitulate the anti-virulence function of native mucin

## References

[1] Bergstrom KSB, Kissoon-Singh V, Gibson DL, Ma C, Montero M, Sham HP, Ryz N, Huang T, Velcich A, Finlay BB, (2010). Muc2 Protects against Lethal Infectious Colitis by Disassociating Pathogenic and Commensal Bacteria from the Colonic Mucosa. PLoS Pathog. 6, e1000902. 10.1371/journal.ppat.1000902. 20485566 PMC2869315

[2] Zarepour M, Bhullar K, Montero M, Ma C, Huang T, Velcich A, Xia L, and Vallance BA (2013). The mucin Muc2 limits pathogen burdens and epithelial barrier dysfunction during Salmonella enterica serovar Typhimurium colitis. Infect. Immun 81, 3672–3683. 10.1128/IAI.00854-13. 23876803 PMC3811786

[3] Atuma C, Strugala V, Allen A, and Holm L (2001). The adherent gastrointestinal mucus gel layer: thickness and physical state in vivo. Am. J. Physiol. Gastrointest. Liver Physiol 280, G922–G929. 10.1152/ajpgi.2001.280.5.G922. 11292601

[4] Ermund A, Schütte A, Johansson MEV, Gustafsson JK, and Hansson GC (2013). Studies of mucus in mouse stomach, small intestine, and colon. I. Gastrointestinal mucus layers have different properties depending on location as well as over the Peyer’s patches. Am. J. Physiol. Gastrointest. Liver Physiol 305, G341–G347. 10.1152/ajpgi.00046.2013. 23832518 PMC3761247

[5] Furter M, Sellin ME, Hansson GC, and Hardt W-D (2019). Mucus Architecture and Near-Surface Swimming Affect Distinct Salmonella Typhimurium Infection Patterns along the Murine Intestinal Tract. Cell Rep. 27, 2665–2678.e3. 10.1016/j.celrep.2019.04.106. 31141690 PMC6547020

[6] Li H, Limenitakis JP, Fuhrer T, Geuking MB, Lawson MA, Wyss M, Brugiroux S, Keller I, Macpherson JA, Rupp S, (2015). The outer mucus layer hosts a distinct intestinal microbial niche. Nat. Commun 6, 8292. 10.1038/ncomms9292. 26392213 PMC4595636

[7] Johansson MEV, Phillipson M, Petersson J, Velcich A, Holm L, and Hansson GC (2008). The inner of the two Muc2 mucin-dependent mucus layers in colon is devoid of bacteria. Proc. Natl. Acad. Sci. USA 105, 15064–15069. 10.1073/pnas.0803124105. 18806221 PMC2567493

[8] Misselwitz B, Barrett N, Kreibich S, Vonaesch P, Andritschke D, Rout S, Weidner K, Sormaz M, Songhet P, Horvath P, (2012). Near Surface Swimming of Salmonella Typhimurium Explains Target-Site Selection and Cooperative Invasion. PLoS Pathog. 8, e1002810. 10.1371/journal.ppat.1002810. 22911370 PMC3406100

[9] Misselwitz B, Kreibich SK, Rout S, Stecher B, Periaswamy B, and Hardt W-D (2011). Salmonella enterica Serovar Typhimurium Binds to HeLa Cells via Fim-Mediated Reversible Adhesion and Irreversible Type Three Secretion System 1-Mediated Docking. Infect. Immun 79, 330–341. 10.1128/iai.00581-10. 20974826 PMC3019867

[10] Lara-Tejero M, and Galán JE (2009). Salmonella enterica serovar typhimurium pathogenicity island 1-encoded type III secretion system translocases mediate intimate attachment to nonphagocytic cells. Infect. Immun 77, 2635–2642. 10.1128/IAI.00077-09. 19364837 PMC2708559

[11] McGhie EJ, Brawn LC, Hume PJ, Humphreys D, and Koronakis V (2009). Salmonella takes control: effector-driven manipulation of the host. Curr. Opin. Microbiol 12, 117–124. 10.1016/j.mib.2008.12.001. 19157959 PMC2647982

[12] Ermund A, Gustafsson JK, Hansson GC, and Keita ÅV (2013). Mucus Properties and Goblet Cell Quantification in Mouse, Rat and Human Ileal Peyer’s Patches. PLoS One 8, e83688. 10.1371/journal.pone.0083688. 24358305 PMC3865249

[13] Hohmann AW, Schmidt G, and Rowley D (1978). Intestinal Colonization and Virulence of Salmonella in Mice. Infect. Immun 22, 763–770. 10.1128/iai.22.3.763-770.1978. 365768 PMC422226

[14] Worley MJ (2025). Salmonella Type III Secretion System Effectors. Int. J. Mol. Sci 26, 2611. 10.3390/ijms26062611. 40141253 PMC11942329

[15] Lou L, Zhang P, Piao R, and Wang Y (2019). Salmonella Pathogenicity Island 1 (SPI-1) and Its Complex Regulatory Network. Front. Cell. Infect. Microbiol 9, 270. 10.3389/fcimb.2019.00270. 31428589 PMC6689963

[16] Lesuffleur T, Barbat A, Dussaulx E, and Zweibaum A (1990). Growth adaptation to methotrexate of HT-29 human colon carcinoma cells is associated with their ability to differentiate into columnar absorptive and mucus-secreting cells. Cancer Res. 50, 6334–6343. 2205381

[17] Augeron C, and Laboisse CL (1984). Emergence of permanently differentiated cell clones in a human colonic cancer cell line in culture after treatment with sodium butyrate. Cancer Res. 44, 3961–3969. 6744312

[18] Swords WE, Cannon BM, and Benjamin WH (1997). Avirulence of LT2 strains of Salmonella typhimurium results from a defective rpoS gene. Infect. Immun 65, 2451–2453. 10.1128/iai.65.6.2451-2453.1997. 9169789 PMC175341

[19] Jarvik T, Smillie C, Groisman EA, and Ochman H (2010). Short-Term Signatures of Evolutionary Change in the Salmonella enterica Serovar Typhimurium 14028 Genome. J. Bacteriol 192, 560–567. 10.1128/jb.01233-09. 19897643 PMC2805332

[20] Wu CM, Wheeler KM, Cárcamo-Oyarce G, Aoki K, McShane A, Datta SS, Mark Welch JL, Tiemeyer M, Griffen AL, and Ribbeck K (2023). Mucin glycans drive oral microbial community composition and function. NPJ Biofilms Microbiomes 9, 11. 10.1038/s41522-023-00378-4. 36959210 PMC10036478

[21] Takagi J, Aoki K, Turner BS, Lamont S, Lehoux S, Kavanaugh N, Gulati M, Valle Arevalo A, Lawrence TJ, Kim CY, (2022). Mucin O-glycans are natural inhibitors of Candida albicans pathogenicity. Nat. Chem. Biol 18, 762–773. 10.1038/s41589-022-01035-1. 35668191 PMC7613833

[22] Ellermeier CD, Ellermeier JR, and Slauch JM (2005). HilD, HilC and RtsA constitute a feed forward loop that controls expression of the SPI1 type three secretion system regulator hilA in Salmonella enterica serovar Typhimurium. Mol. Microbiol 57, 691–705. 10.1111/j.1365-2958.2005.04737.x. 16045614

[23] Lim S, Yun J, Yoon H, Park C, Kim B, Jeon B, Kim D, and Ryu S (2007). Mlc regulation of Salmonella pathogenicity island I gene expression via hilE repression. Nucleic Acids Res. 35, 1822–1832. 10.1093/nar/gkm060. 17329372 PMC1874608

[24] Baxter MA, and Jones BD (2005). The fimYZ Genes Regulate Salmonella enterica Serovar Typhimurium Invasion in Addition to Type 1 Fimbrial Expression and Bacterial Motility. Infect. Immun 73, 1377–1385. 10.1128/IAI.73.3.1377-1385.2005. 15731035 PMC1064959

[25] Joiner JD, Steinchen W, Mozer N, Kronenberger T, Bange G, Poso A, Wagner S, and Hartmann MD (2023). HilE represses the activity of the Salmonella virulence regulator HilD via a mechanism distinct from that of intestinal long-chain fatty acids. J. Biol. Chem 299, 105387. 10.1016/j.jbc.2023.105387. 37890783 PMC10696396

[26] Schechter LM, and Lee CA (2001). AraC/XylS family members, HilC and HilD, directly bind and derepress the Salmonella typhimurium hilA promoter. Mol. Microbiol 40, 1289–1299. 10.1046/j.1365-2958.2001.02462.x. 11442828

[27] Cooper KG, Chong A, Kari L, Jeffrey B, Starr T, Martens C, McClurg M, Posada VR, Laughlin RC, Whitfield-Cargile C, (2021). Regulatory protein HilD stimulates Salmonella Typhimurium invasiveness by promoting smooth swimming via the methyl-accepting chemotaxis protein McpC. Nat. Commun 12, 348. 10.1038/s41467-020-20558-6. 33441540 PMC7806825

[28] Chowdhury R, Pavinski Bitar PD, Keresztes I, Condo AM Jr., and Altier C (2021). A diffusible signal factor of the intestine dictates Salmonella invasion through its direct control of the virulence activator HilD. PLoS Pathog. 17, e1009357. 10.1371/journal.ppat.1009357. 33617591 PMC7932555

[29] Yang X, Stein KR, and Hang HC (2023). Anti-infective bile acids bind and inactivate a Salmonella virulence regulator. Nat. Chem. Biol 19, 91–100. 10.1038/s41589-022-01122-3. 36175659 PMC9805502

[30] Eade CR, Hung C-C, Bullard B, Gonzalez-Escobedo G, Gunn JS, and Altier C (2016). Bile Acids Function Synergistically To Repress Invasion Gene Expression in Salmonella by Destabilizing the Invasion Regulator HilD. Infect. Immun 84, 2198–2208. 10.1128/iai.00177-16. 27185788 PMC4962646

[31] Wu Y, Yang X, Zhang D, and Lu C (2020). Myricanol Inhibits the Type III Secretion System of Salmonella enterica Serovar Typhimurium by Interfering With the DNA-Binding Activity of HilD. Front. Microbiol 11, 571217. 10.3389/fmicb.2020.571217. 33101243 PMC7546796

[32] Hung C-C, Garner CD, Slauch JM, Dwyer ZW, Lawhon SD, Frye JG, McClelland M, Ahmer BMM, and Altier C (2013). The intestinal fatty acid propionate inhibits almonella invasion through the post-translational control of. Mol. Microbiol 87, 1045–1060. 10.1111/mmi.12149. 23289537 PMC3581741

[33] Du Z, Su H, Wang W, Ye L, Wei H, Peng Z, Anishchenko I, Baker D, and Yang J (2021). The trRosetta server for fast and accurate protein structure prediction. Nat. Protoc 16, 5634–5651. 10.1038/s41596-021-00628-9. 34759384

[34] Meller A, Ward M, Borowsky J, Kshirsagar M, Lotthammer JM, Oviedo F, Ferres JL, and Bowman GR (2023). Predicting locations of cryptic pockets from single protein structures using the PocketMiner graph neural network. Nat. Commun 14, 1177. 10.1038/s41467-023-36699-3. 36859488 PMC9977097

[35] Midgett CR, Talbot KM, Day JL, Munson GP, and Kull FJ (2021). Structure of the master regulator Rns reveals an inhibitor of enterotoxigenic Escherichia coli virulence regulons. Sci. Rep 11, 15663. 10.1038/s41598-021-95123-2. 34341412 PMC8329261

[36] Trott O, and Olson AJ (2010). AutoDock Vina: Improving the speed and accuracy of docking with a new scoring function, efficient optimization, and multithreading. J. Comput. Chem 31, 455–461. 10.1002/jcc.21334. 19499576 PMC3041641

[37] Eberhardt J, Santos-Martins D, Tillack AF, and Forli S (2021). Auto-Dock Vina 1.2.0: New Docking Methods, Expanded Force Field, and Python Bindings. J. Chem. Inf. Model 61, 3891–3898. 10.1021/acs.jcim.1c00203. 34278794 PMC10683950

[38] Taylor ME, Bezouska K, and Drickamer K (1992). Contribution to ligand binding by multiple carbohydrate-recognition domains in the macrophage mannose receptor. J. Biol. Chem 267, 1719–1726. 1730714

[39] Kiessling LL, Young T, Gruber TD, and Mortell KH (2008). Multivalency in Protein–Carbohydrate Recognition. In Glycoscience: Chemistry and Chemical Biology, Fraser-Reid BO, Tatsuta K, and Thiem J, eds. (Springer), pp. 2483–2523. 10.1007/978-3-540-30429-6_64.

[40] Liang R, Loebach J, Horan N, Ge M, Thompson C, Yan L, and Kahne D (1997). Polyvalent binding to carbohydrates immobilized on an insoluble resin. Proc. Natl. Acad. Sci. USA 94, 10554–10559. 10.1073/pnas.94.20.10554. 9380673 PMC23398

[41] Kramer JR, Onoa B, Bustamante C, and Bertozzi CR (2015). Chemically tunable mucin chimeras assembled on living cells. Proc. Natl. Acad. Sci. USA 112, 12574–12579. 10.1073/pnas.1516127112. 26420872 PMC4611660

[42] Deleray AC, and Kramer JR (2022). Biomimetic Glycosylated Polythreonines by N-Carboxyanhydride Polymerization. Biomacromolecules 23, 1453–1461. 10.1021/acs.biomac.2c00020. 35104406

[43] Deming TJ (1999). Cobalt and Iron Initiators for the Controlled Polymerization of α-Amino Acid-N-Carboxyanhydrides. Macromolecules 32, 4500–4502. 10.1021/ma9902899.

[44] Wood AM, Wardzala CL, and Kramer JR (2025). Chemoenzymatic synthesis of sialylated and fucosylated mucin analogs reveals glycan-dependent effects on protein conformation and degradation. RSC Chem. Biol 6, 1336–1352. 10.1039/D5CB00111K. 40677303 PMC12266245

[45] Raev SA, Amimo JO, Saif LJ, and Vlasova AN (2023). Intestinal mucin-type O-glycans: the major players in the host-bacteria-rotavirus interactions. Gut Microbes 15, 2197833. 10.1080/19490976.2023.2197833. 37020288 PMC10078158

[46] Baos SC, Phillips DB, Wildling L, McMaster TJ, and Berry M (2012). Distribution of Sialic Acids on Mucins and Gels: A Defense Mechanism. Biophys. J 102, 176–184. 10.1016/j.bpj.2011.08.058. 22225812 PMC3250687

[47] Ma X, Li M, Wang X, Qi G, Wei L, and Zhang D (2024). Sialylation in the gut: From mucosal protection to disease pathogenesis. Carbohydr. Polym 343, 122471. 10.1016/j.carbpol.2024.122471. 39174097

[48] Bergstrom K, Fu J, Johansson MEV, Liu X, Gao N, Wu Q, Song J, McDaniel JM, McGee S, Chen W, (2017). Core 1- and 3-derived O-glycans collectively maintain the colonic mucus barrier and protect against spontaneous colitis in mice. Mucosal Immunol. 10, 91–103. 10.1038/mi.2016.45. 27143302 PMC5097036

[49] Diard M, Garcia V, Maier L, Remus-Emsermann MNP, Regoes RR, Ackermann M, and Hardt W-D (2013). Stabilization of cooperative virulence by the expression of an avirulent phenotype. Nature 494, 353–356. 10.1038/nature11913. 23426324

[50] Sturm A, Heinemann M, Arnoldini M, Benecke A, Ackermann M, Benz M, Dormann J, and Hardt W-D (2011). The Cost of Virulence: Retarded Growth of Salmonella Typhimurium Cells Expressing Type III Secretion System 1. PLoS Pathog. 7, e1002143. 10.1371/journal.ppat.1002143. 21829349 PMC3145796

[51] Brinkkötter A, Klöss H, Alpert C, and Lengeler JW (2000). Pathways for the utilization of N-acetyl-galactosamine and galactosamine in Escherichia coli. Mol. Microbiol 37, 125–135. 10.1046/j.1365-2958.2000.01969.x. 10931310

[52] Arabyan N, Weis AM, Huang BC, and Weimer BC (2017). Implication of Sialidases in Salmonella Infection: Genome Release of Sialidase Knockout Strains from Salmonella enterica Serovar Typhimurium LT2. Genome Announc. 5, e00341–17. 10.1128/genomea.00341-17. 28495784 PMC5427219

[53] Arabyan N, Park D, Foutouhi S, Weis AM, Huang BC, Williams CC, Desai P, Shah J, Jeannotte R, Kong N, (2016). Salmonella Degrades the Host Glycocalyx Leading to Altered Infection and Glycan Remodeling. Sci. Rep 6, 29525. 10.1038/srep29525. 27389966 PMC4937416

[54] Hoyer LL, Hamilton AC, Steenbergen SM, and Vimr ER (1992). Cloning, sequencing and distribution of the Salmonella typhimurium LT2 sialidase gene, nanH, provides evidence for interspecies gene transfer. Mol. Microbiol 6, 873–884. 10.1111/j.1365-2958.1992.tb01538.x. 1602967

[55] Hoyer LL, Roggentin P, Schauer R, and Vimr ER (1991). Purification and properties of cloned Salmonella typhimurium LT2 sialidase with virustypical kinetic preference for sialyl alpha 2-—3 linkages. J. Biochem 110, 462–467. 10.1093/oxfordjournals.jbchem.a123603. 1769974

[56] Perez de la Cruz Moreno M, Oth M, Deferme S, Lammert F, Tack J, Dressman J, and Augustijns P (2006). Characterization of fasted-state human intestinal fluids collected from duodenum and jejunum. J. Pharm. Pharmacol 58, 1079–1089. 10.1211/jpp.58.8.0009. 16872555

[57] Cummings JH, Pomare EW, Branch WJ, Naylor CP, and Macfarlane GT (1987). Short chain fatty acids in human large intestine, portal, hepatic and venous blood. Gut 28, 1221–1227. 10.1136/gut.28.10.1221. 3678950 PMC1433442

[58] Johansson MEV, Larsson JMH, and Hansson GC (2011). The two mucus layers of colon are organized by the MUC2 mucin, whereas the outer layer is a legislator of host-microbial interactions. Proc. Natl. Acad. Sci. USA 108, 4659–4665. 10.1073/pnas.1006451107. 20615996 PMC3063600

[59] Robbe C, Capon C, Coddeville B, and Michalski J-C (2004). Structural diversity and specific distribution of O-glycans in normal human mucins along the intestinal tract. Biochem. J 384, 307–316. 10.1042/BJ20040605. 15361072 PMC1134114

[60] Robbe C, Capon C, Maes E, Rousset M, Zweibaum A, Zanetta J-P, and Michalski J-C (2003). Evidence of regio-specific glycosylation in human intestinal mucins: presence of an acidic gradient along the intestinal tract. J. Biol. Chem 278, 46337–46348. 10.1074/jbc.M302529200. 12952970

[61] Li X, Bleumink-Pluym NMC, Luijkx YMCA, Wubbolts RW, van Putten JPM, and Strijbis K (2019). MUC1 is a receptor for the Salmonella SiiE adhesin that enables apical invasion into enterocytes. PLoS Pathog. 15, e1007566. 10.1371/journal.ppat.1007566. 30716138 PMC6375660

[62] Schubert C, Nguyen BD, Sichert A, Näpflin N, Sintsova A, Feer L, Näf J, Daniel BBJ, Steiger Y, von Mering C, (2025). Monosaccharides drive Salmonella gut colonization in a context-dependent or -independent manner. Nat. Commun 16, 1735. 10.1038/s41467-025-56890-y. 39966379 PMC11836396

[63] Wang BX, Takagi J, McShane A, Park JH, Aoki K, Griffin C, Teschler J, Kitts G, Minzer G, Tiemeyer M, (2023). Host-derived O-glycans inhibit toxigenic conversion by a virulence-encoding phage in Vibrio cholerae. EMBO J. 42, e111562. 10.15252/embj.2022111562. 36504455 PMC9890226

[64] Wheeler KM, Cárcamo-Oyarce G, Turner BS, Dellos-Nolan S, Co JY, Lehoux S, Cummings RD, Wozniak DJ, and Ribbeck K (2019). Mucin glycans attenuate the virulence of Pseudomonas aeruginosa in infection. Nat. Microbiol 4, 2146–2154. 10.1038/s41564-019-0581-8. 31611643 PMC7157942

[65] Werlang CA, Chen WG, Aoki K, Wheeler KM, Tymm C, Mileti CJ, Burgos AC, Kim K, Tiemeyer M, and Ribbeck K (2021). Mucin O-glycans suppress quorum-sensing pathways and genetic transformation in Streptococcus mutans. Nat. Microbiol 6, 574–583. 10.1038/s41564-021-00876-1. 33737747 PMC8811953

[66] Caldara M, Friedlander RS, Kavanaugh NL, Aizenberg J, Foster KR, and Ribbeck K (2012). Mucin biopolymers prevent bacterial aggregation by retaining cells in the free-swimming state. Curr. Biol 22, 2325–2330. 10.1016/j.cub.2012.10.028. 23142047 PMC3703787

[67] Co JY, Cárcamo-Oyarce G, Billings N, Wheeler KM, Grindy SC, Holten-Andersen N, and Ribbeck K (2018). Mucins trigger dispersal of Pseudomonas aeruginosa biofilms. npj Biofilms Microbiomes 4, 1–8. 10.1038/s41522-018-0067-0. 30323945 PMC6180003

[68] Kavanaugh NL, Zhang AQ, Nobile CJ, Johnson AD, and Ribbeck K (2014). Mucins suppress virulence traits of Candida albicans. mBio 5, e01911. 10.1128/mBio.01911-14. 25389175 PMC4235211

[69] Wang BX, Wheeler KM, Cady KC, Lehoux S, Cummings RD, Laub MT, and Ribbeck K (2021). Mucin Glycans Signal through the Sensor Kinase RetS to Inhibit Virulence-Associated Traits in Pseudomonas aeruginosa. Curr. Biol 31, 90–102.e7. 10.1016/j.cub.2020.09.088. 33125866 PMC8759707

[70] Ohneck EJ, Arivett BA, Fiester SE, Wood CR, Metz ML, Simeone GM, and Actis LA (2018). Mucin acts as a nutrient source and a signal for the differential expression of genes coding for cellular processes and virulence factors in Acinetobacter baumannii. PLoS One 13, e0190599. 10.1371/journal.pone.0190599. 29309434 PMC5757984

[71] Lange MD, Farmer BD, and Abernathy J (2018). Catfish mucus alters the Flavobacterium columnare transcriptome. FEMS Microbiol. Lett 365, fny244. 10.1093/femsle/fny244. 30285236

[72] Fancy N, Inaba R, Kniffen D, Kniffen D, Melvin M, Kazemian N, Sa- deghi J, D’Aloisio L, D’Aloisio L, Copp AG, (2024). Fecal-adherent mucus is a non-invasive source of primary human MUC2 for structural and functional characterization in health and disease. J. Biol. Chem 300, 105675. 10.1016/j.jbc.2024.105675. 38272223 PMC10891339

[73] Venkatakrishnan V, Quintana-Hayashi MP, Mahu M, Haesebrouck F, Pasmans F, and Lindén SK (2017). Brachyspira hyodysenteriae Infection Regulates Mucin Glycosylation Synthesis Inducing an Increased Expression of Core-2 O-Glycans in Porcine Colon. J. Proteome Res 16, 1728–1742. 10.1021/acs.jproteome.7b00002. 28301166

[74] Lerminiaux NA, MacKenzie KD, and Cameron ADS (2020). Salmonella Pathogenicity Island 1 (SPI-1): The Evolution and Stabilization of a Core Genomic Type Three Secretion System. Microorganisms 8, 576. 10.3390/microorganisms8040576. 32316180 PMC7232297

[75] Hautefort I, Proença MJ, and Hinton JCD (2003). Single-copy green fluorescent protein gene fusions allow accurate measurement of Salmonella gene expression in vitro and during infection of mammalian cells. Appl. Environ. Microbiol 69, 7480–7491. 10.1128/AEM.69.12.7480-7491.2003. 14660401 PMC310007

[76] Datsenko KA, and Wanner BL (2000). One-step inactivation of chromosomal genes in Escherichia coli K-12 using PCR products. Proc. Natl. Acad. Sci. USA 97, 6640–6645. 10.1073/pnas.120163297. 10829079 PMC18686

[77] Sharan SK, Thomason LC, Kuznetsov SG, and Court DL (2009). Recombineering: a homologous recombination-based method of genetic engineering. Nat. Protoc 4, 206–223. 10.1038/nprot.2008.227. 19180090 PMC2790811

[78] Cherepanov PP, and Wackernagel W (1995). Gene disruption in Escherichia coli: TcR and KmR cassettes with the option of Flp-catalyzed excision of the antibiotic-resistance determinant. Gene 158, 9–14. 10.1016/0378-1119(95)00193-a. 7789817

[79] Detwiler RE, McPartlon TJ, Coffey CS, and Kramer JR (2023). Clickable Polyprolines from Azido-proline N-Carboxyanhydride. ACS Polym. Au 3, 383–393. 10.1021/acspolymersau.3c00011. 37841952 PMC10571246

[80] Clauss ZS, Wardzala CL, Schlirf AE, Wright NS, Saini SS, Onoa B, Bustamante C, and Kramer JR (2021). Tunable, biodegradable grafting-from glycopolypeptide bottlebrush polymers. Nat. Commun 12, 6472. 10.1038/s41467-021-26808-5. 34753949 PMC8578664

[81] Astre G, Créchet L, Pomié N, Pereira O, Cani PD, Knauf C, and Abot A (2024). Protocol for the preclinical evaluation of gut barrier function and immune interaction in an HT-29/PBMC co-culture model. STAR Protoc. 5, 103416. 10.1016/j.xpro.2024.103416. 39487982 PMC11565454

[82] Galaxy Community (2024). The Galaxy platform for accessible, reproducible, and collaborative data analyses: 2024 update. Nucleic Acids Res. 52, W83–W94. 10.1093/nar/gkae410. 38769056 PMC11223835

[83] Li H, and Durbin R (2009). Fast and accurate short read alignment with Burrows–Wheeler transform. Bioinformatics 25, 1754–1760. 10.1093/bioinformatics/btp324. 19451168 PMC2705234

[84] Anders S, Pyl PT, and Huber W (2015). HTSeq—a Python framework to work with high-throughput sequencing data. Bioinformatics 31, 166–169. 10.1093/bioinformatics/btu638. 25260700 PMC4287950

[85] Love MI, Huber W, and Anders S (2014). Moderated estimation of fold change and dispersion for RNA-seq data with DESeq2. Genome Biol. 15, 550. 10.1186/s13059-014-0550-8. 25516281 PMC4302049

[86] Chen H. (2022). VennDiagram: Generate High-Resolution Venn and Euler Plots. Version 1.7.3.https://CRAN.R-project.org/package=VennDiagram

[87] Wu T, Hu E, Xu S, Chen M, Guo P, Dai Z, Feng T, Zhou L, Tang W, Zhan L, (2021). clusterProfiler 4.0: A universal enrichment tool for interpreting omics data. Innovation 2, 100141. 10.1016/j.xinn.2021.100141. 34557778 PMC8454663

[88] Jumper J, Evans R, Pritzel A, Green T, Figurnov M, Ronneberger O, Tunyasuvunakool K, Bates R, Žídek A, Potapenko A, (2021). Highly accurate protein structure prediction with AlphaFold. Nature 596, 583–589. 10.1038/s41586-021-03819-2. 34265844 PMC8371605

[89] Lin Z, Akin H, Rao R, Hie B, Zhu Z, Lu W, Smetanin N, Verkuil R, Kabeli O, Shmueli Y, (2023). Evolutionary-scale prediction of atomic-level protein structure with a language model. Science 379, 1123–1130. 10.1126/science.ade2574. 36927031

[90] Studer G, Rempfer C, Waterhouse AM, Gumienny R, Haas J, and Schwede T (2020). QMEANDisCo—distance constraints applied on model quality estimation. Bioinformatics 36, 1765–1771. 10.1093/bioinformatics/btz828. 31697312 PMC7075525

[91] Kirschner KN, Yongye AB, Tschampel SM, González-Outeiriño J, Daniels CR, Foley BL, and Woods RJ (2008). GLYCAM06: A generalizable biomolecular force field. J. Comput. Chem 29, 622–655. 10.1002/jcc.20820. 17849372 PMC4423547

[92] Zhang Y, Forli S, Omelchenko A, and Sanner MF (2019). AutoGridFR: Improvements on AutoDock Affinity Maps and Associated Software Tools. J. Comput. Chem 40, 2882–2886. 10.1002/jcc.26054. 31436329 PMC7737998

[93] Meng EC, Goddard TD, Pettersen EF, Couch GS, Pearson ZJ, Morris JH, and Ferrin TE (2023). UCSF ChimeraX: Tools for structure building and analysis. Protein Sci. 32, e4792. 10.1002/pro.4792. 37774136 PMC10588335

[94] Zhang ZJ, Pedicord VA, Peng T, and Hang HC (2020). Site-specific acylation of a bacterial virulence regulator attenuates infection. Nat. Chem. Biol 16, 95–103. 10.1038/s41589-019-0392-5. 31740807 PMC8439376

