# Supplementary material for: Mucus-derived glycans are inhibitory signals for Salmonella Typhimurium SPI-1-mediated invasion
Source: Cell Rep. Author manuscript; Available in PMC 2026 May 15. (PMC13179021; doi:10.1016/j.celrep.2025.116304)
Supplement: Supplemental Material [file NIHMS2170014-supplement-Supplemental_Material.pdf]

Cell Reports, Volume *44*

Supplemental information

Mucus-derived glycans are inhibitory signals for *Salmonella* Typhimurium SPI-1-mediated invasion

Kelsey M. Wheeler, Michaela A. Gold, Corey A. Stevens, Karsten Tedin, Amanda M. Wood, Deniz Uzun, Gerardo Cárcamo-Oyarce, Bradley S. Turner, Marcus Fulde, Jeongmin Song, Jessica R. Kramer, and Katharina Ribbeck

Supplementary Information:

Document S1. Figures S1-S6, Tables S1-S3

Figures:

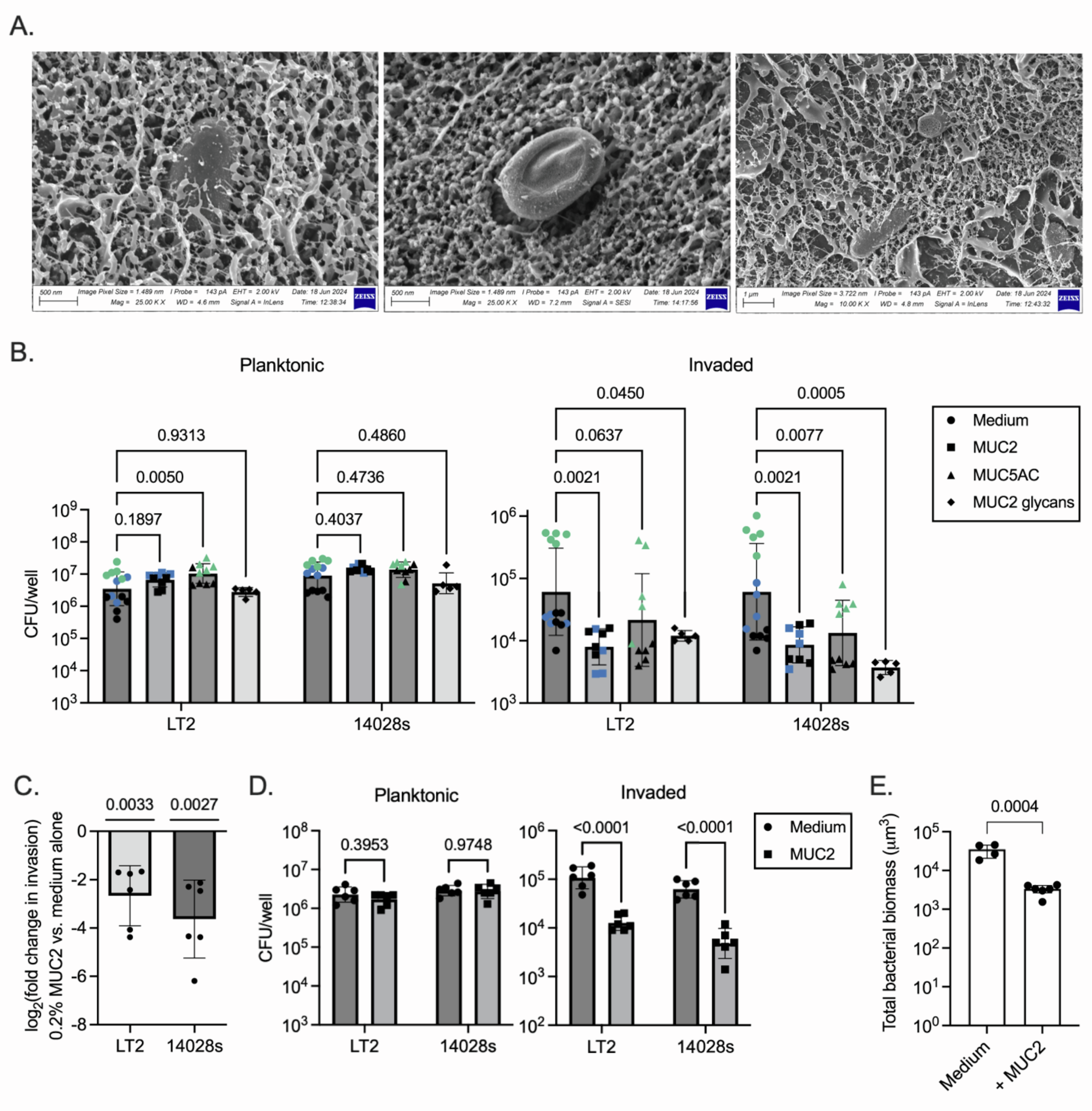

Figure S1. The intestinal mucin MUC2 is an anti-infective signal.

(A) Representative cryogenic scanning electron microscopy images of *S*. Typhimurium grown with purified MUC2 (0.4% w/v). The left panel is the raw image for **Figure 1A.**

(B) Effect of purified porcine MUC2 (0.2% w/v), MUC5AC (0.2% w/v) or MUC2 glycans (0.05% w/v) on *S*. Typhimurium invasion of polarized HT-29 cells (MOI = 20), as determined by enumerating total bacterial CFU (left) and the invaded bacterial cells that survived gentamicin treatment (right). Data points represent individual biological replicates; sets of experiments that were completed in parallel are indicated by color. These CFUs are the underlying data for **Figures 1B, 1C, and 3B**. Bars represent mean $\log_{10}$-adjusted CFU, and error bars represent the SD. A two-way ANOVA with Šídák's multiple comparisons test was conducted to evaluate whether there was a significant difference in bacterial CFU in medium alone relative to medium with mucin or glycans. Exact *p* values reported.

(C) Host-cell invasion, as determined by a gentamicin protection assay of *S*. Typhimurium grown with purified porcine MUC2 (0.2% w/v), of polarized HT-29 cells cultured on transwells and infected at a

multiplicity of infection (MOI) of 20. Data points represent individual biological replicates, bars represent mean log_2-adjusted changes relative to medium alone, and error bars represent the SD. A one-sample *t*-test was performed to evaluate whether the change in invasion was significantly different than 0. Exact *p* values reported.

(D) Effect of purified porcine MUC2 (0.2% w/v) on *S*. Typhimurium invasion (MOI = 20) of polarized HT-29 cells cultured on Transwells, as determined by enumerating total bacterial CFU (left), and the invaded bacterial cells that survived gentamicin treatment (right). Data points represent individual biological replicates, bars represent mean log_{10}-adjusted CFU, and error bars represent the SD. A two-way ANOVA with Šídák's multiple comparisons test was conducted to evaluate whether there was a significant difference in bacterial CFU in medium alone relative to medium with MUC2. Exact *p* values reported. These data are the underlying CFU for **Figure S1C**.

(E) Quantification of *Salmonella* invasion (MOI = 200) in medium with or without purified MUC2 (0.2% w/v) for the invasion assays presented in **Figure 1D**. Data points represent the calculated volume of bacterial biomass based on fluorescent intensity for different frames, the bar represents the median biomass, and the error bars represent the interquartile range. An unpaired t-test was conducted to evaluate whether there was a significant difference in biomass in medium alone relative to medium with MUC2. Exact *p* values reported.

See also Figures 1 and 3.

A.

Downregulated Pathway	P-value
Carbon metabolism	1.26E-05
Glyoxylate and dicarboxylate metabolism	1.29E-04
***Salmonella* infection**	**1.29E-04**
Propanoate metabolism	9.39E-04
Citrate cycle (TCA cycle)	0.00609
Microbial metabolism in diverse environments	0.00623
**Upregulated Pathway**	**P-value**
Glycolysis / Gluconeogenesis	1.68E-07
Phosphotransferase system (PTS)	2.18E-04
Methane metabolism	4.95E-04
Fructose and mannose metabolism	6.32E-04
Microbial metabolism in diverse environments	9.96E-04
Carbon metabolism	0.00107

B.

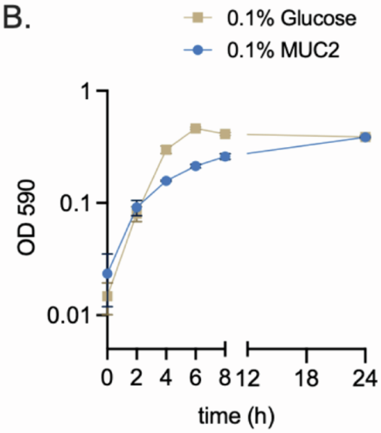

C.

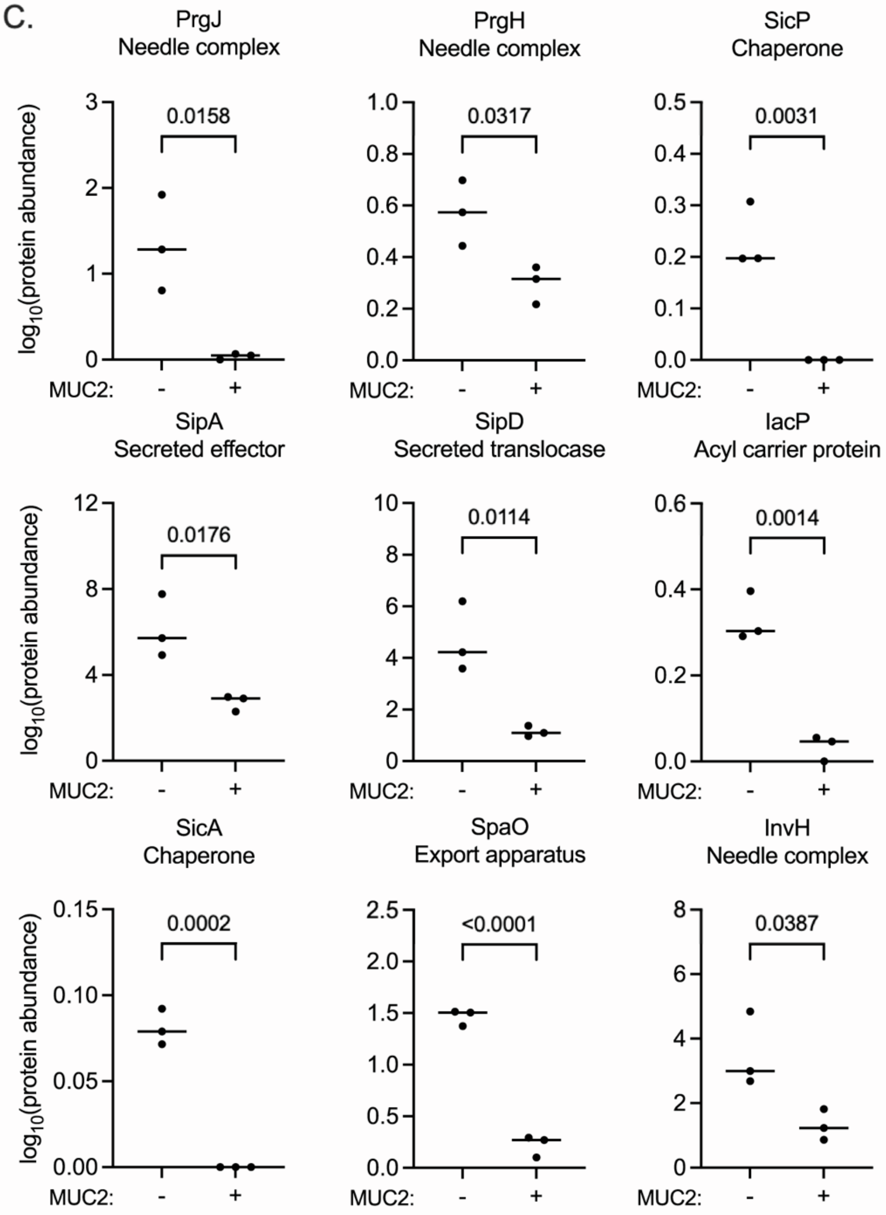

Figure S2. The intestinal mucin MUC2 is an anti-infective signal.

(A) Enrichment of metabolic and infection-related KEGG pathways in differentially regulated gene sets. P-values are adjusted for multiple comparisons. The table includes all pathways with $P_{adjusted}<0.01$.

(B) Path-length adjusted optical density of *S*. Typhimurium in 100 µL M9 culture medium supplemented with 0.1% glucose or 0.1% MUC2 as the sole carbon source. Data points represent the mean absorbance of n = 3 replicates, and the error bars represent the SD.

(C) *Salmonella* protein expression in medium with or without purified MUC2 (0.1% w/v) for the nine SPI-1 proteins identified by data-independent acquisition proteomics. Data points represent the log_{10}-adjusted protein abundance, and the central line represents the median protein abundance for the n = 3 biological replicates. An unpaired t-test was conducted to evaluate whether there was a significant difference in biomass in medium alone relative to medium with MUC2. Exact *p* values reported.

See also Figure 2.

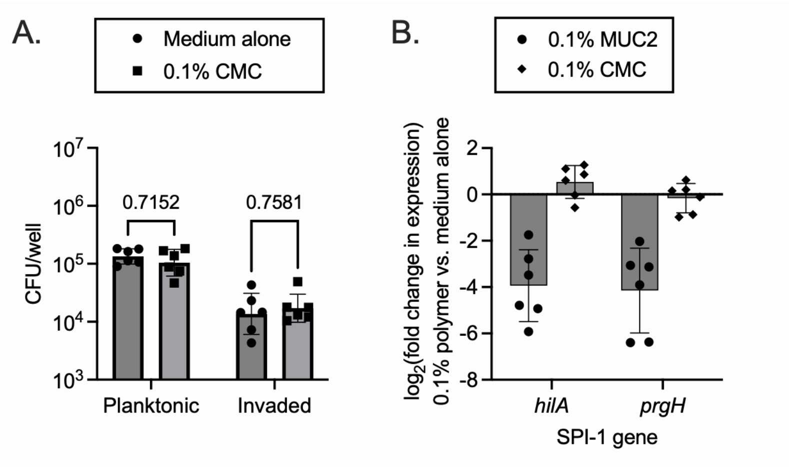

Figure S3. Carboxymethyl cellulose is not sufficient to alter host cell invasion or SPI-1 expression for *S.* Typhimurium.

(A) Effect of carboxymethyl cellulose (0.1% w/v) on *S*. Typhimurium invasion (MOI = 20) of HT-29 cells, as determined by enumerating total bacterial CFU (left), and the invaded bacterial cells that survived gentamicin treatment (right). Data points represent individual biological replicates, bars represent mean $\log_{10}$-adjusted CFU, and error bars represent the SD. A two-way ANOVA with Šídák's multiple comparisons test was conducted to evaluate whether there was a significant difference in bacterial CFU in medium alone relative to medium with CMC. Exact *p* values reported.

(B) Effect of carboxymethyl cellulose versus MUC2 (0.1% w/v) on SPI-1 gene expression, as measured by qPCR.

See also Figures 1 and 2.

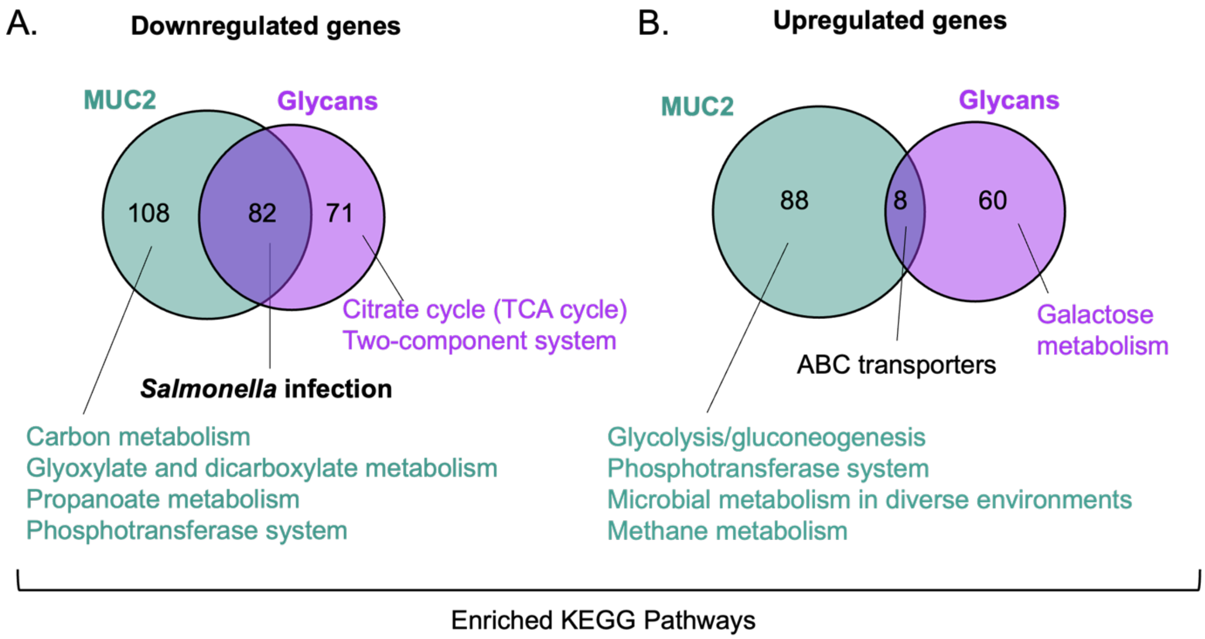

Figure S4. The intestinal mucin MUC2 is an anti-infective signal.

(A) Venn diagrams of downregulated gene sets for MUC2- and MUC2-glycan-treated cells, showing enriched metabolic and infection-related KEGG pathways.

(B) Venn diagrams of upregulated gene sets for MUC2- and MUC2-glycan-treated cells, showing enriched KEGG pathways.

See also Figure 3.

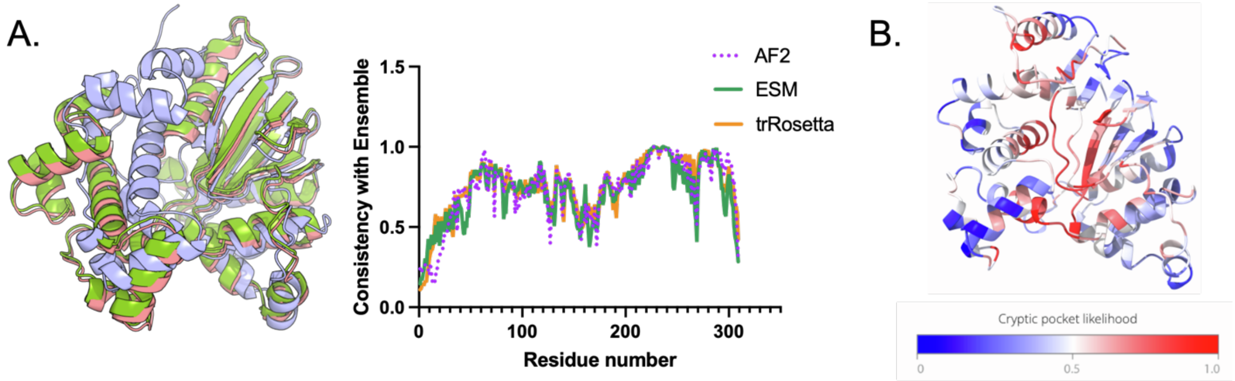

Figure S5. MUC2-derived sugars inhibit *S*. Typhimurium virulence via the HilD ligand binding pocket.

(A) Overlay of the predicted 3D structures of HilD from Alphafold2 (red), trRosetta (blue), and ESM (green) (left). QMEAN calculated per-residue consistency between structures (right).

(B) Cryptic binding pocket prediction for the trRosetta 3D model of HilD. Potential binding pockets on the predicted HilD structure were identified with PocketMiner based on size, shape, hydrophobicity, and potential hydrogen-bonding interactions. Red color indicates higher likelihood.

See also Figure 4C.

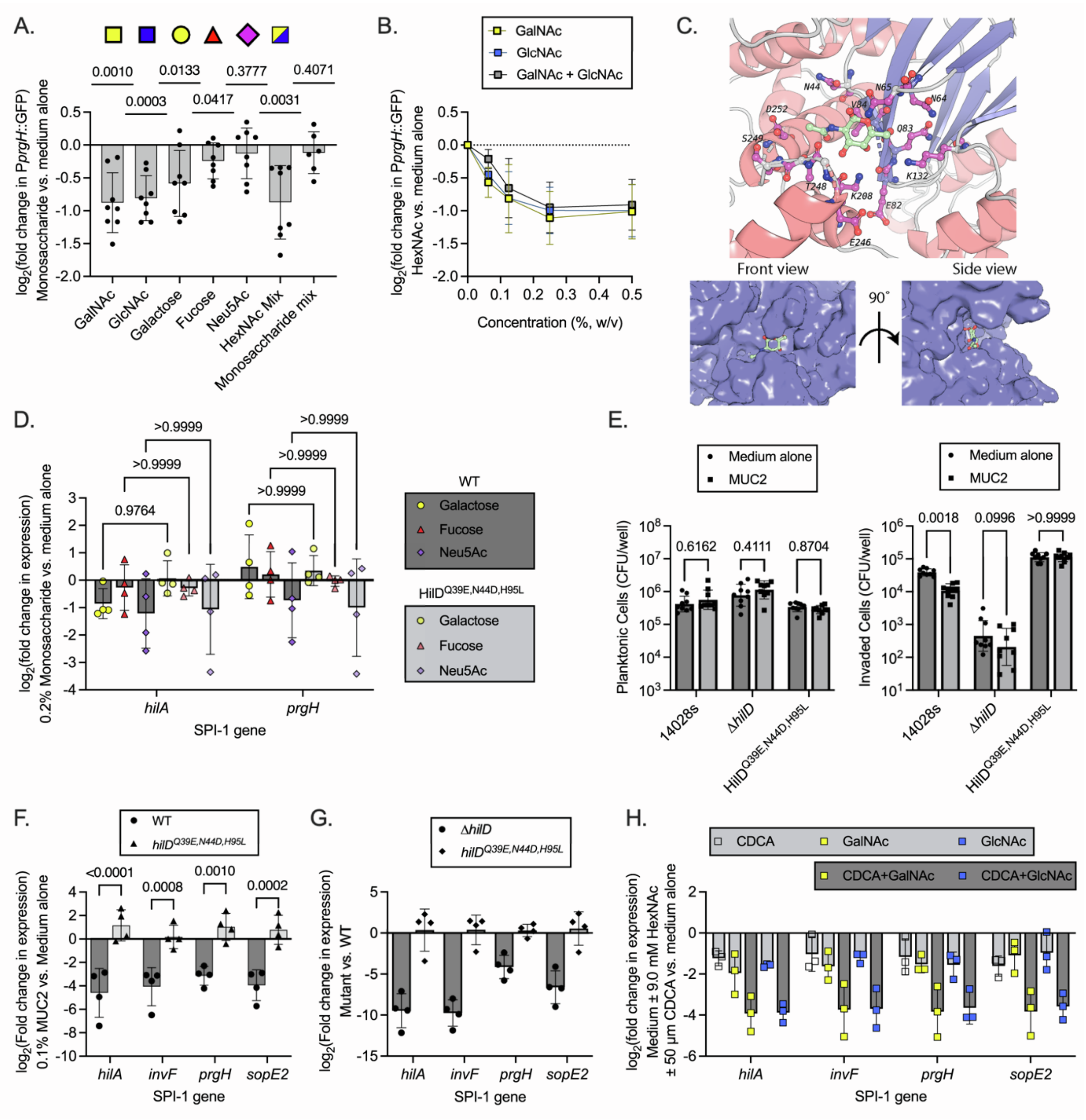

Figure S6. MUC2-derived sugars inhibit *S.* Typhimurium virulence via the HilD ligand binding pocket.

(A) Screening SPI-suppression by individual monosaccharides using a reporter for *prgH* transcriptional activity (*S.* Typhimurium 14028s PprgH::GFP). Data points represent individual biological replicates, bars represent mean $\log_2$-adjusted changes relative to medium alone, and error bars represent the SD. A one-sample *t*-test was performed to evaluate whether the change in expression was significantly different than 0. Exact *p*-values are shown above each bar.

(B) Dose-response of prgH-GFP reporter activity to increasing concentrations of GalNAc, GlcNAc, and a combination of GalNAc + GlcNAc. Concentrations refer to total monosaccharide content, i.e., for the mixed HexNAc condition, 0.5% corresponds to 0.25% GalNAc (~11 mM) + 0.25% GlcNAc (~11 mM). Data points represent the mean change in fluorescence across n = 6 biological replicates, with error bars showing the SD.

(C) Top: Predicted binding pose of GalNAc within the putative HilD ligand-binding pocket. Binding location and pose were visualized using PyMOL. Highlighted residues indicate amino acids within

5 angstroms of the potential GalNAc binding position. Bottom: 3D-filled model of GalNAc monomers docking within the predicted HilD ligand binding pocket.

(D) Effect of HilD binding site mutations on SPI-1 gene expression, measured by qPCR, in response to 0.2% w/v galactose (11 mM), fucose (12.2 mM), or sialic acid (6.5 mM). Data points represent individual biological replicates, bars represent mean log_2-adjusted changes relative to medium alone, and error bars represent the SD. A two-way ANOVA with Tukey's multiple comparison test was conducted to evaluate the effect of HilD mutations on monosaccharide-mediated changes of SPI-1 gene expression. Exact *p* values reported.

(E) Effect of purified porcine MUC2 (0.2% w/v) on *S*. Typhimurium WT or HilD mutant invasion (MOI = 20) of HT-29 cells, as determined by enumerating total bacterial CFU (left), and the invaded bacterial cells that survived gentamicin treatment (right). Data points represent individual biological replicates, bars represent mean log_{10}-adjusted CFU, and error bars represent the SD. A two-way ANOVA with Šídák's multiple comparisons test was conducted to evaluate whether there was a significant difference in bacterial CFU in medium alone relative to medium with MUC2. Exact *p* values reported.

(F) Effect of HilD binding site mutations on SPI-1 gene expression, measured by qPCR, in response to purified MUC2 (0.1% w/v). Data points represent individual biological replicates, bars represent mean log_2-adjusted changes relative to medium alone, and error bars represent the SD. A two-way ANOVA with Šídák's multiple comparisons test was conducted to evaluate the effect of the HilD mutation on MUC2-mediated suppression of SPI-1 gene expression. Exact *p* values reported.

(G) The *hilD* knockout mutant has reduced SPI-1 expression, measured by qPCR, while the signal binding domain mutant has WT-level expression of SPI-1. Data points represent individual biological replicates, bars represent mean log_2-adjusted changes relative to medium alone, and error bars represent the SD.

(H) Chenodeoxycholic acid and HexNAc have additive effects on SPI-1 downregulation. SPI-1 gene expression with or without GalNAc or GlcNAc (concentration = 0.2% w/v) and the bile acid, CDCA (50 μM), measured by qPCR. Data points represent individual biological replicates, the bar represents the mean log_2-adjusted change in expression relative to medium alone, and the error bars represent the standard deviation.

See also Figure 4.

Tables:

Table S1. MUC2 glycan structures identified by MS and the corresponding proposed structure(s).

Obs m/z	Delta Mass	Percent	Composition	Core	Proposed Structure(s)
447.2222	0.002	1.64%	(Hex)$_1$ (Deoxyhexose)$_1$		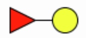
477.2331	0.001	0.78%	(Hex)$_2$		
518.26	0.002	2.37%	(Hex)$_1$ (HexNAc)$_1$	Core 1	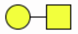
559.2869	0.002	0.46%	(HexNAc)$_2$	Core 3	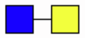
634.3085	0.003	18.99%	(Hex)$_1$ (NeuAc)$_1$		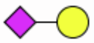
664.3194	0.004	9.67%	(Hex)$_1$ (NeuGc)$_1$		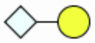
675.3355	0.004	2.00%	(HexNAc)$_1$ (NeuAc)$_1$	Core 1	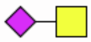
681.3351	0.004	0.18%	(Hex)$_3$		
692.3511	0.004	35.56%	(Hex)$_1$ (HexNAc)$_1$ (Deoxyhexose)$_1$	Core 1	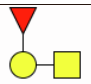
705.3464	0.004	0.52%	(HexNAc)$_1$ (NeuGc)$_1$	Core 1	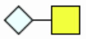
722.3619	0.004	0.70%	(Hex)$_2$ (HexNAc)$_1$	Core 1	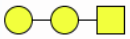
763.3887	0.005	1.90%	(Hex)$_1$ (HexNAc)$_2$	Core 1	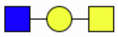
804.4158	0.005	0.10%	(HexNAc)$_3$	Core 3	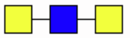
879.4374	0.006	6.05%	(Hex)$_1$ (HexNAc)$_1$ (NeuAc)$_1$	Core 1	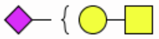
896.4529	0.006	0.02%	(Hex)$_2$ (HexNAc)$_1$ (Deoxyhexose)$_1$	Core 1	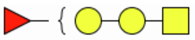
909.4483	0.007	2.98%	(Hex)$_1$ (HexNAc)$_1$ (NeuGc)$_1$	Core 1	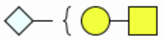
920.4644	0.007	2.11%	(HexNAc)$_2$ (NeuAc)$_1$	Core 3	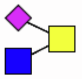
926.4644	0.007	0.06%	(Hex)$_3$ (HexNAc)$_1$	Core 1	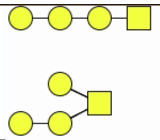
937.4797	0.006	5.34%	(Hex)$_1$ (HexNAc)$_2$ (Deoxyhexose)$_1$	Core 1 and Core 3	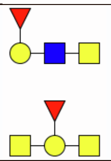
950.4751	0.006	0.81%	(HexNAc)$_2$ (NeuGc)$_1$	Core 3	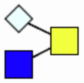
967.4906	0.007	0.57%	(Hex)$_2$ (HexNAc)$_2$	Core 1	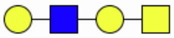
1008.518	0.007	0.52%	(Hex)$_1$ (HexNAc)$_3$	Core 1	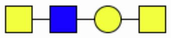

1053.529	0.008	0.65%	$(Hex)_1$ $(HexNAc)_1$ $(Deoxyhexose)_1$ $(NeuAc)_1$	Core 1	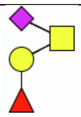
1070.545	0.009	0.05%	$(Hex)_2$ $(HexNAc)_1$ $(Deoxyhexose)_2$	Core 1	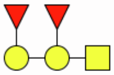
1083.539	0.008	0.24%	(Hex)1 (HexNAc)1 (Deoxyhexose) 1 (NeuGc)1 and (Hex)2 (HexNAc)1 (NeuAc)1	Core 1	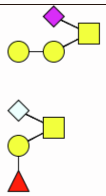
1089.539	0.008	0.06%	$(Hex)_5$		
1111.571	0.008	0.05%	$(Hex)_1$ $(HexNAc)_2$ $(Deoxyhexose)_2$	Core 3	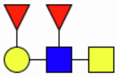
1113.551	0.009	0.16%	$(Hex)_2$ $(HexNAc)_1$ $(NeuGc)_1$	Core 1	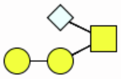
1124.564	0.006	0.77%	$(Hex)_1$ $(HexNAc)_2$ $(NeuAc)_1$	Core 1 and Core 3	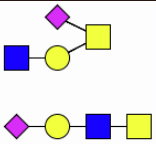
1130.567	0.009	0.02%	$(Hex)_4$ $(HexNAc)_1$		Possible N-glycan; Paucimannose
1154.574	0.006	0.39%	$(Hex)_1$ $(HexNAc)_2$ $(NeuGc)_1$	Core 1 and Core 3	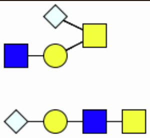
1171.593	0.009	0.09%	$(Hex)_3$ $(HexNAc)_2$	Core 1	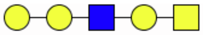
1182.606	0.006	0.77%	$(Hex)_1$ $(HexNAc)_3$ $(Deoxyhexose)_1$	Core 1 and Core 3	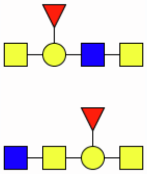
1212.617	0.006	0.22%	$(Hex)_2$ $(HexNAc)_3$	Core 1 and Core 3	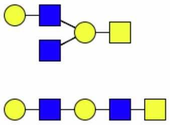
1240.612	0.007	0.84%	$(Hex)_1$ $(HexNAc)_1$ $(NeuAc)_2$	Core 1	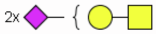
1253.6475	0.011	0.02%	$(Hex)_1$ $(HexNAc)_4$	Core 2	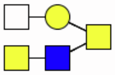
1270.623	0.007	0.13%	$(Hex)_1$ $(HexNAc)_1$ $(NeuAc)_1$ $(NeuGc)_1$	Core 1	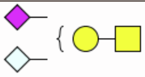
1298.655	0.008	0.21%	$(Hex)_1$ $(HexNAc)_2$ $(Deoxyhexose)_1$ $(NeuAc)_1$	Core 1	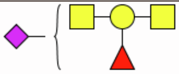
1300.634	0.007	0.06%	$(Hex)_1$ $(HexNAc)_1$ $(NeuGc)_2$	Core 1	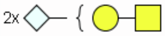

1315.672	0.01	0.19%	(Hex)$_2$ (HexNAc)$_2$ (Deoxyhexose)$_2$	Core 1	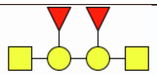
1328.665	0.008	0.25%	(Hex)$_2$ (HexNAc)$_2$ (NeuAc)$_1$ and (Hex)$_1$ (HexNAc)$_2$ (Deoxyhexose)$_1$ (NeuGc)$_1$	Core 1 and Core 3	Multiple structures
1345.684	0.012	0.06%	(Hex)$_3$ (HexNAc)$_2$ (Deoxyhexose)$_1$	Core 1	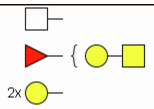
1358.68	0.012	0.10%	(Hex)$_2$ (HexNAc)$_2$ (NeuGc)$_1$	Core 1	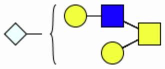
1375.695	0.012	0.00%	(Hex)$_4$ (HexNAc)$_2$	NA	Most likely N-glycan; Man$_4$
1386.708	0.008	0.16%	(Hex)$_2$ (HexNAc)$_3$ (Deoxyhexose)$_1$	Core 1	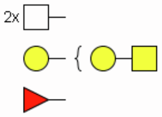
1416.719	0.009	0.10%	(Hex)$_3$ (HexNAc)$_3$	NA	Most likely N-glycan; Complex Type
1427.739	0.013	0.14%	(Hex)$_1$ (HexNAc)$_4$ (Deoxyhexose)$_1$	Core 2	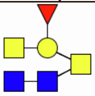
1485.741	0.009	0.03%	(Hex)$_1$ (HexNAc)$_2$ (NeuAc)$_2$	Core 1 and Core 3	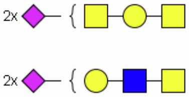
1502.761	0.015	0.03%	(Hex)$_2$ (HexNAc)$_2$ (Deoxyhexose)$_1$ (NeuAc)$_1$	Core 1	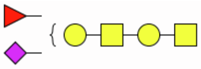
1532.772	0.015	0.02%	(Hex)$_3$ (HexNAc)$_2$ (NeuAc)$_1$ and (Hex)$_2$ (HexNAc)$_2$ (Deoxyhexose)$_1$ (NeuGc)$_1$	Core 1	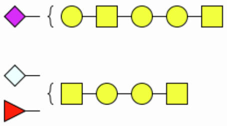
1543.787	0.014	0.08%	(Hex)$_1$ (HexNAc)$_3$ (Deoxyhexose)$_1$ (NeuAc)$_1$	Core 3	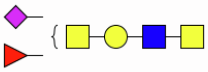
1560.803	0.014	0.13%	(Hex)$_2$ (HexNAc)$_3$ (Deoxyhexose)$_2$	Core 2	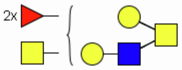
1573.794	0.01	0.04%	(Hex)$_1$ (HexNAc)$_3$ (Deoxyhexose)$_1$ (NeuGc)$_1$ and (Hex)$_2$ (HexNAc)$_3$ (NeuAc)$_1$	Core 1 and Core 3	Multiple glycoforms
1579.794	0.01	0.41%	(Hex)$_5$ (HexNAc)$_2$	NA	Most likely an N-glycan; Man$_5$
1590.815	0.015	0.03%	(Hex)$_3$ (HexNAc)$_3$ (Deoxyhexose)$_1$	NA	Most likely an N-glycan; Core fucosylated Complex
1620.821	0.011	0.09%	(Hex)$_4$ (HexNAc)$_3$	NA	Most likely an N-glycan; Complex
1631.837	0.011	0.04%	(Hex)$_2$ (HexNAc)$_4$ (Deoxyhexose)$_1$	Core 2	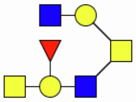
1661.847	0.011	0.06%	(Hex)$_3$ (HexNAc)$_4$		Most likely an N-glycan; Complex

Table S2. PCR primers used in this study.

Primer	Gene	Primer Sequence (5´ > 3´)
k1	*kan*(*aph*)	CAGTCATAGCCGAATAGCCT
k2	*kan*(*aph*)	CGGTGCCCTGAATGAACTGC
FIMZF	*fimZ*	TTAAGGTGTCTGACGCTTAT
FIMZR	*fimZ*	ATCAATTACAATTAGTGTCC
FIMZH1P1	*fimZ*	CGCTTATTATAAAACGAAGGACGCATAACAGTCTGAGGCATACAACAAT GTGTAGGCTGGAGCTGCTTCGA
FIMZH2P2	*fimZ*	TGTGGCTCCCGAACGATAATTCGCCGGGAGTACATTTACAATAATTCGT GCATATGAATATCCTCCTTAG
HILEF	*hilE*	GGCAGAAGGTATTTAGCAAG
HILER	*hilE*	TGTAAGGGCCACGCGTTATC
HILEH1P1	*hilE*	GATTGTCGGTATTTAATCTGGTATACAGAGACACCAACGAAATGTGTAG GCTGGAGCTGCTTCGA
HILEH3P2	*hilE*	CAGCATCGCCCACTGCGAGTCCGCAAGCTTGTTTTGTCCTCATCGCATA TGAATATCCTCCTTAG
MLCF	*mlc*	TGTGCAGTTAATCACACGGC
MLCR	*mlc*	GATATGGCAAGGGCAATCAG
MLCH1P1	*mlc*	CATAGCACACAGATTATTTCGGAGCGCGAAAATAAAGGGAGTGAGCGG TGTGTAGGCTGGAGCTGCTTCGA
MLCH2P2	*mlc*	GCTTGAGTTAGCGCAAATTTTTGTACAACAGTTAAAAAATGTTAACCCTG CATATGAATATCCTCCTTAG

Table S3. qPCR primers used in this study.

Primer	Sequence
gyrB-F	TACGGAAACCACCGCAATCA
gyrB-R	GGTACTCACCTTGCAGGCTT
hilA-F	GGGCAGATGATACCCGATGG
hilA-R	AAGAGAGAAGCGGGTTGGTG
prgH-F	GCCAGTGCTGCTACAATTCC
prgH-R	TACGCCAATACAGGTCGGTG
sopE2-F	CCTTTTGTCGTCCCCCTCAT
sopE2-R	AAATTGTTGTGGCGTTGGCA
invF-F	ATGGCGCAGGATTAGTGGAC
invF-R	ACTCGCAGCGTTTACGATCT
